# Functional dissection of the C-terminal domain of rabies virus RNA polymerase L protein

**DOI:** 10.1128/jvi.02082-24

**Published:** 2025-03-11

**Authors:** Fumiki Izumi, Machiko Makino, Michihito Sasaki, Kento Nakagawa, Tatsuki Takahashi, Shoko Nishiyama, Yuji Fujii, Misuzu Okajima, Tatsunori Masatani, Manabu Igarashi, Hirofumi Sawa, Makoto Sugiyama, Naoto Ito

**Affiliations:** 1 Joint Graduate School of Veterinary Sciences, Gifu University 12785 , Gifu, Japan; 2 Research Fellow of Japan Society for the Promotion of Science (JSPS), Tokyo, Japan; 3 Laboratory of Zoonotic Diseases, Faculty of Applied Biological Sciences, Gifu University 98331 , Gifu, Japan; 4 Division of Molecular Pathobiology, International Institute for Zoonosis Control, Hokkaido University 12810 , Sapporo, Japan; 5 Institute for Vaccine Research and Development (IVReD), Hokkaido University 12810 , Sapporo, Japan; 6 The United Graduate School of Veterinary Sciences, Gifu University 12785 , Gifu, Japan; 7 Center for One Medicine Innovative Translational Research (COMIT), Gifu University 12785 , Gifu, Japan; 8 Division of Global Epidemiology, International Institute for Zoonosis Control, Hokkaido University 12810 , Sapporo, Japan; University Medical Center Freiburg, Freiburg, Germany

**Keywords:** rabies, RNA polymerases

## Abstract

The rabies virus large (L) protein interacts with its cofactor phosphoprotein (P protein) to function as an RNA-dependent RNA polymerase (RdRp). The C-terminal domain (CTD) of the L protein plays a critical role in P protein binding. We previously reported that the highly conserved NPYNE sequence in the hydrophilic region of the CTD (positions 1929–1933 of the L protein [L1929–1933]) is important for both P protein binding and RdRp function. To elucidate the functional role of the CTD in detail, we examined the importance of each of the hydrophilic residues in the NPYNE sequence (underlined). A rabies virus mutant with Ala substitutions in these hydrophilic residues showed low replication capacity. Comprehensive analyses of a revertant of the mutant virus and a series of L protein mutants revealed that Asn at L1929 is crucial for both P protein binding and RdRp function. Analyses of the L protein mutants also showed that Asn at L1932 and Glu at L1933 have roles in RdRp function and P protein binding, respectively. Furthermore, we demonstrated that the NPYNE sequence is essential for stabilizing the L protein through the L-P interaction. In a previously determined L protein structure, all of the hydrophilic residues in the NPYNE sequence form the first α-helix in the CTD. Therefore, our findings indicate that this helix is important for P protein-binding ability, RdRp function, and stabilization of the L protein, thereby contributing to efficient viral replication.

**IMPORTANCE:**

Although RNA-dependent RNA polymerase of rhabdoviruses, which is composed of the large (L) protein and its cofactor phosphoprotein (P protein), has a high potential as a target for therapeutics against the viruses, the relationship between the structure and molecular functions is poorly understood. In this study, we functionally examined the C-terminal domain (CTD) of the rabies virus L protein as a model for the rhabdovirus L protein. We showed that the first α-helix in the CTD is important for the P protein-binding ability, RdRp function, and stability of the L protein. Since in the L-P complex structure, this helix does not form an interface with the P protein, we provide here the first evidence of an indirect contribution of the L protein CTD to the L-P interaction in rhabdoviruses. The findings in this study will be useful for developing therapeutics targeting the L-P interaction.

## INTRODUCTION

Viruses belonging to the family *Rhabdoviridae* in the order *Mononegavirales* include various human pathogenic viruses causing fatal encephalitis, such as rabies virus (RABV) and Chandipura virus (CHPV). RABV (*Lyssavirus rabies*), which is classified into the genus *Lyssavirus*, is a causative agent of rabies, a lethal neurological disease with a case fatality rate of almost 100% in humans and animals. It is estimated that approximately 59,000 people die of this disease every year, mainly in developing countries in Africa and Asia (1). Meanwhile, CHPV (*Vesiculovirus chandipura*), which is classified into the genus *Vesiculovirus*, is an emerging human pathogen that causes an acute encephalitis syndrome with a high case fatality rate, mostly in children. This virus is endemic in India and regularly causes outbreaks mainly in rural areas (2–7). Since no therapy has been established for these diseases, there is an urgent need for the development of therapeutics against these human pathogenic rhabdoviruses.

The large (L) protein of rhabdovirus, which is known as an RNA-dependent RNA polymerase (RdRp), is a promising target for the development of therapeutics against the virus since this protein plays essential and pivotal roles in viral replication by effecting its multiple enzymatic functions. Specifically, the L protein participates in the transcription and modification of viral mRNAs through its transcriptase, capping, methyltransferase, and polyadenylation enzyme activities (8–12). The L protein also conducts the replication of viral genomic RNA via its replicase activity (8). Theoretically, inhibition of these L protein functions would result in negative impacts on the biosynthesis of all viral components, including viral genomic RNA and proteins, and thus on the production of progeny viruses.

Rhabdovirus L protein requires physical interaction with its essential cofactor phosphoprotein (P protein) to properly function as an RdRp. Specifically, the P protein bridges the L protein with the nucleoprotein (N protein) that enwraps genomic RNA to form the ribonucleoprotein complex, which acts as an active template both for mRNA transcription and genome replication (8). In addition to this necessary function in viral RNA synthesis, the P protein of the vesicular stomatitis virus (VSV), which represents a virus belonging to the family *Rhabdoviridae*, has a function to stabilize the L protein molecule through their interaction (13), although this function has remained to be examined in the RABV P protein. These facts strongly suggest that the L-P interaction plays multiple essential roles in viral RNA synthesis and thus has a high potential as a target for the development of antiviral therapeutics that strongly inhibit L protein functions. To develop therapeutics targeting the L-P interaction, it is necessary to understand the relation between molecular functions and structures of the L-P complex.

**Fig 1:**
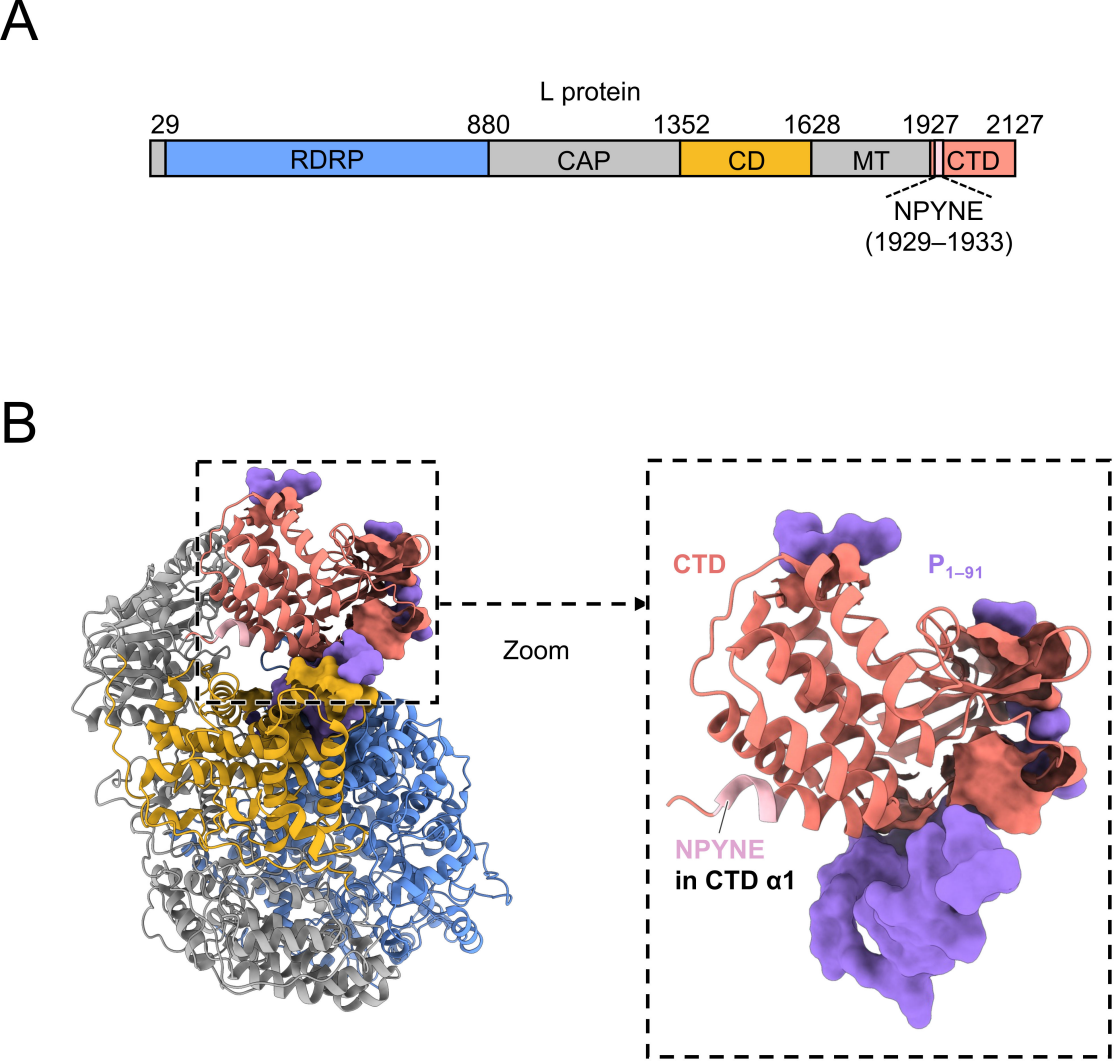
Location of the NPYNE sequence in the RABV L protein. (**A**) Domain organization of the RABV L protein. Domains containing the residues that directly interact with the P protein (RDRP, CD, and CTD) are colored blue, yellow, and red, respectively. (**B**) Structure of the RABV L protein in complex with P_1–91_ (PDB ID: 6UEB). The L protein is shown in cartoon representation and is colored by domains corresponding to panel A. The L protein residues directly interacting with the P protein are shown with their molecular surfaces. P_1–91_ is shown in surface representation and is colored purple. The dashed square indicates the zoomed view of the L protein CTD and P_1–91_.

Recent structural analyses using cryogenic electron microscopy revealed the L protein structures of RABV and VSV in complex with the N-terminal fragment of the P protein (14, 15), providing structural insights into the molecular functions of the L proteins. Similar to the L proteins of other viruses in the order *Mononegavirales* (16–26), these L proteins are composed of three functional domains with enzymatic active sites for RNA syntheses and mRNA maturation (RdRp domain [RDRP], capping domain [CAP], and methyltransferase domain [MT]) and two structural domains without enzymatic activity (connector domain [CD] and C-terminal domain [CTD]) (14, 15, 27, 28). Furthermore, these structures illustrated the binding mode of the P protein to the L protein; the P protein fragment wraps around the CTD of the L protein and binds with the RDRP, CD, and CTD (14, 15) (Fig. 1A and B). Although this suggests the importance of the CTD in the L-P interaction, it is largely unknown how this non-enzymatic domain contributes to L protein functions.

We previously analyzed the molecular function of an RABV L protein as a model for the rhabdovirus L protein and obtained findings highlighting the functional importance of the CTD. Specifically, we found that the NPYNE sequence at positions 1929–1933 of the L protein (L1929–1933), which is located in the N-terminal side of the CTD (14) (Fig. 1A), is important for both the P protein-binding ability and RdRp function (29). This is supported by the findings that substitutions of three hydrophilic residues in the NPYNE sequence (underlined) with hydrophobic Ala residues (APYAA) greatly reduced the L protein functions, indicating the functional importance of the three hydrophilic residues. These findings strongly suggest that the CTD has an important role in RdRp function through the formation of the L-P complex.

Since the RABV L protein binds exclusively with the N-terminal portion of the P protein (30, 31), the previously reported structure of the RABV L protein complexed with the N-terminal fragment of the P protein spanning residues 1–91 (P_1–91_) (14) should reflect the whole aspect of the binding between L and P proteins. In this structure, the NPYNE sequence is located at the N-terminus of the first α-helix that constitutes the CTD, CTD α1 (Fig. 1B), where there is no interface for the P protein binding, indicating that the NPYNE sequence is involved in the L-P interaction in an indirect manner. The NPYNE sequence is located in an amino acid region that is highly functionally constrained and almost completely conserved among lyssavirus species (29) (Fig. S1), suggesting that the CTD α1 including this sequence plays an important and universal role in the L-P interaction in these species. However, the mechanism by which the CTD indirectly contributes to the L-P interaction remains to be elucidated.

In this study, to elucidate the role of the CTD of the RABV L protein in the L-P interaction, we examined the functional importance of each hydrophilic residue in the NPYNE sequence (underlined) located at L1929–1933. First, by a reverse genetics approach, we generated an RABV mutant in which the NPYNE sequence at L1929–1933 was substituted with an APYAA sequence and then confirmed that this substitution reduced viral replication capacity. Next, by serial passages of the RABV mutant, we obtained a revertant virus that overcomes the replication deficiency caused by the APYAA substitution. Analyses using the revertant virus and various L protein mutants showed that Asn at position 1929 in the L protein (L1929) plays important roles in both its P protein-binding ability and RdRp function. Furthermore, we demonstrated that the L-P interaction regulated by the CTD is important for the stabilization of the L protein molecule. These findings suggest that the CTD α1, including Asn at L1929, of the RABV L protein plays indispensable, multiple roles in P protein binding, RdRp function, and L protein stabilization by indirectly contributing to the L-P interaction and thus will be useful information for developing therapeutics targeting the interaction.

## RESULTS

### Establishment and characterization of a RABV mutant with APYAA substitution at L1929–1933

**Fig 2:**
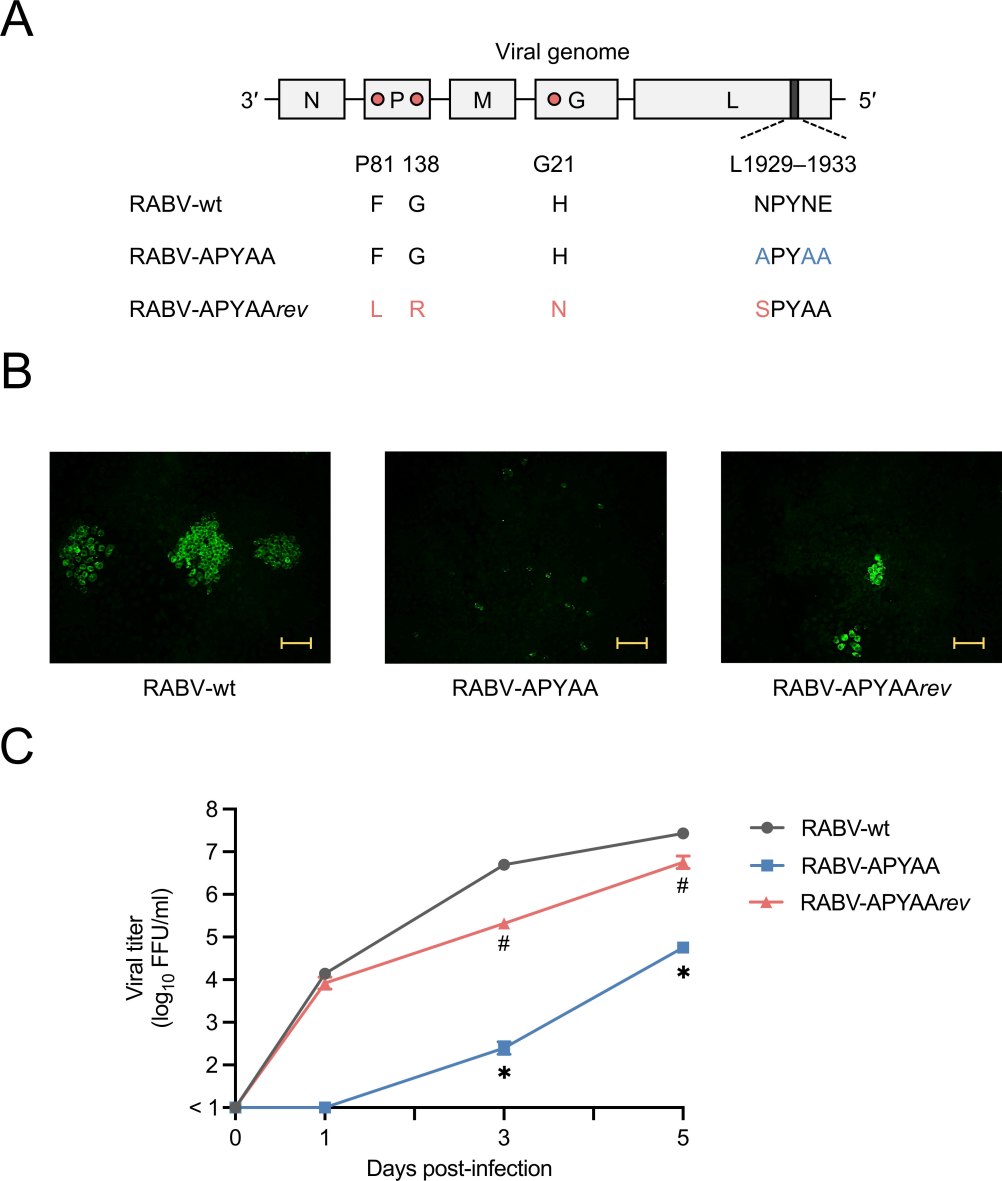
Replication capacity of RABV-wt, RABV-APYAA, and RABV-APYAA*rev*. (**A**) Schematic diagrams of the genome organization of RABV-wt, RABV-APYAA, and RABV-APYAA*rev*. Gray boxes represent open reading frames derived from RABV-wt. A dark gray box represents the position of the NPYNE sequence. Pink circles indicate the amino acid residues where mutations were observed in RABV-APYAA*rev*. Blue and red letters indicate the amino acid residues that differ between RABV-wt and RABV-APYAA and between RABV-APYAA and RABV-APYAA*rev*, respectively. (**B**) Cell-to-cell spread of RABV-wt, RABV-APYAA, and RABV-APYAA*rev*. NA cells were infected with each virus at a multiplicity of infection (MOI) of 0.01. At 2 d.p.i., the cells were fixed and immunostained with an anti-RABV N protein mouse monoclonal antibody. The yellow bars indicate 100 µm. (**C**) Growth of RABV-wt, RABV-APYAA, and RABV-APYAA*rev*. NA cells were infected with each virus at an MOI of 0.01, and the culture supernatants were harvested at 0, 1, 3, and 5 d.p.i. Virus titers in the culture supernatants were determined by a focus assay. The values in the graph are means ± standard error (SE) of the means. *, significant difference between RABV-APYAA and the other two viruses (*P* < 0.05); #, significant difference between RABV-APYAA*rev* and the other two viruses (*P* < 0.05).

We previously reported that the substitution of the three hydrophilic residues in the NPYNE sequence at L1929–1933 with hydrophobic Ala residues (APYAA) substantially reduced the P protein binding ability and the RdRp function (29). This indicates the possibility that the APYAA substitution decreases viral replication capacity and thus virulence through the reduced L-P interaction. To examine the possibility, we attempted to establish an RABV mutant that has the APYAA sequence instead of the NPYNE sequence at L1929–1933 by using a reverse genetics system of the pathogenic RABV Nishigahara strain (RABV-wt) (32). Although it was expected that the APYAA substitution would severely reduce the RdRp function of the L protein (29), we successfully rescued the mutant, which we named RABV-APYAA (Fig. 2A), indicating that the substitution does not completely ruin viral viability.

**Fig 3:**
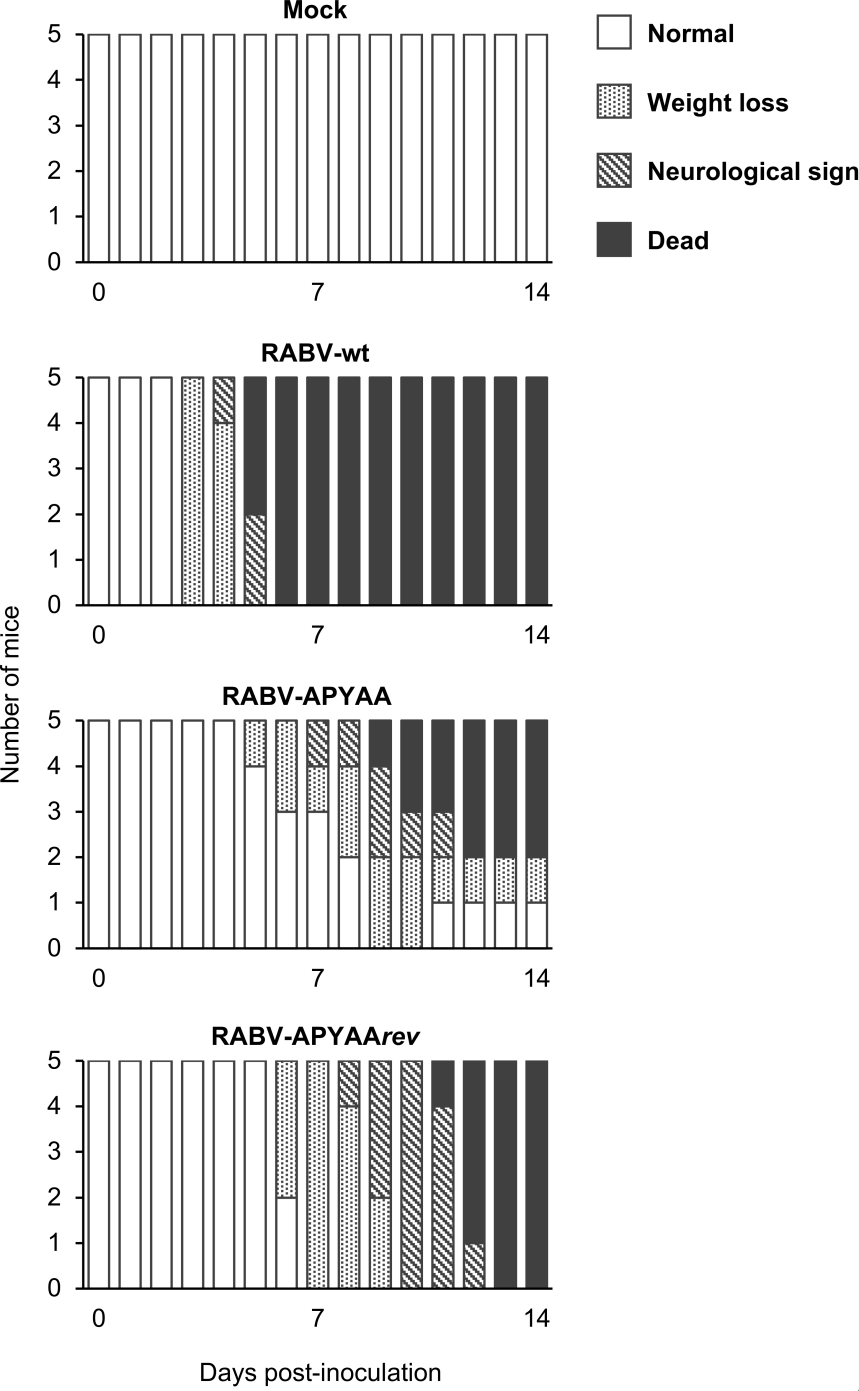
Virulence of RABV-wt, RABV-APYAA, and RABV-APYAA*rev* in mice. Five 6-week-old male ddY mice were inoculated with 10^3^ focus-forming units of RABV-wt, RABV-APYAA, or RABV-APYAA*rev* via the intracerebral route. Mock-infected mice were inoculated with a diluent. Each mouse was observed daily for 14 days to record body weight, clinical signs, and mortality.

To examine the replication capacity of RABV-APYAA, we assessed its cell-to-cell spread and growth in mouse neuroblastoma C1300 (NA) cells. Compared to RABV-wt, RABV-APYAA formed substantially smaller foci (Fig. 2B) and grew with significantly lower efficiency (Fig. 2C). These results demonstrate that the replication capacity of RABV-APYAA is significantly lower than that of RABV-wt. Next, to check whether the replication deficiency of RABV-APYAA negatively affects its virulence, we inoculated mice intracerebrally with RABV-APYAA. None of the RABV-wt-inoculated mice survived, whereas 40% of the RABV-APYAA-inoculated mice tolerated the infection (Fig. 3), indicating that the virulence of RABV-APYAA is lower than that of RABV-wt. These results demonstrate that the APYAA substitution reduces the replication capacity and virulence of an RABV strain, indicating that the L-P interaction is important both for its replication and virulence.

### Biological and genetic characterization of the RABV-APYAA revertant

To obtain molecular information on the RABV L-P interaction, we sought to establish an RABV-APYAA revertant that overcomes the replication deficiency caused by the APYAA substitution at L1929–1933 and then to identify mutations that compensate for the functional defect of the RABV-APYAA L protein. First, we serially passaged RABV-APYAA in NA cells 20 times and obtained RABV-APYAA*rev* after cloning of the passaged virus. Compared to RABV-APYAA, RABV-APYAA*rev* formed larger foci (Fig. 2B) and grew more efficiently (Fig. 2C), indicating that the replication capacity of RABV-APYAA*rev* is higher than that of RABV-APYAA. Furthermore, unlike RABV-APYAA, RABV-APYAA*rev* killed all of the infected mice (Fig. 3), indicating that RABV-APYAA*rev* is more virulent than RABV-APYAA. These findings indicate that RABV-APYAA*rev* can be considered as a revertant of RABV-APYAA.

Next, to identify the amino acid mutations that occurred during the serial passages of RABV-APYAA, we genetically analyzed RABV-APYAA and RABV-APYAA*rev* stocks by next-generation sequencing. Most of the RABV-APYAA populations (>99%) retained the APYAA sequence at L1929–1933 and no unexpected mutation was observed (Table 1). We found that RABV-APYAA*rev* acquired a total of four amino acid mutations after the passages: more than 99% of the populations acquired Phe-to-Leu/Gly-to-Arg mutations at positions 81/138 in the P protein (P81/138), a His-to-Asn mutation at position 21 in the glycoprotein (G protein), and an Ala-to-Ser mutation at L1929 (Table 1; Fig. 2A).

**TABLE 1:** Genetic analysis of RABV-APYAA and RABV-APYAA*rev*

Position		Strain				
Nucleotide (Nt)^*a*^	Protein amino acid (AA)	RABV-wt (reference)	RABV-APYAA		RABV-APYAA*rev*	
		Nt (AA)	Nt (AA)^*b*^	Proportion (%)	Nt (AA)^*b*^	Proportion (%)
1063	N-331	C (Ala)			T (syn)	99.8
1066	N-332	A (Ala)			G (syn)	99.7
1755	P-81	T (Phe)			**C (Leu**)	98.6
1926	P-138	G (Gly)			**A (Arg**)	99.7
3434	G-21	C (His)			**A (Asn**)	> 99.9
5212	Non-coding	A (-)			G (-)	99.8
7665	L-752	T (Leu)	C (Leu)	28.9		
8114	L-901	G (Val)			A (Val)	97.9
10976	L-1855	G (Gly)			A (Gly)	99.4
11196–11198	L-1929	AAT (Asn)	**GCA (Ala**)	99.6	**TCA (Ser**)	99.3
11205–11207	L-1932	AAT (Asn)	**GCA (Ala**)	99.8	**GCA (Ala**)	99.5
11209–11210	L-1933	AG (Glu)	**CT (Ala**)	99.8	**CT (Ala**)	99.8

^*a*^ Based on genome nucleotide number of RABV-wt (GenBank accession number: AB044824).

^*b*^ Nucleotide changes involving amino acid substitutions are shown in bold.

### Examination of compensatory effects of the mutations at P81/138 and L1929 on the functional defect of the L protein

**Fig 4:**
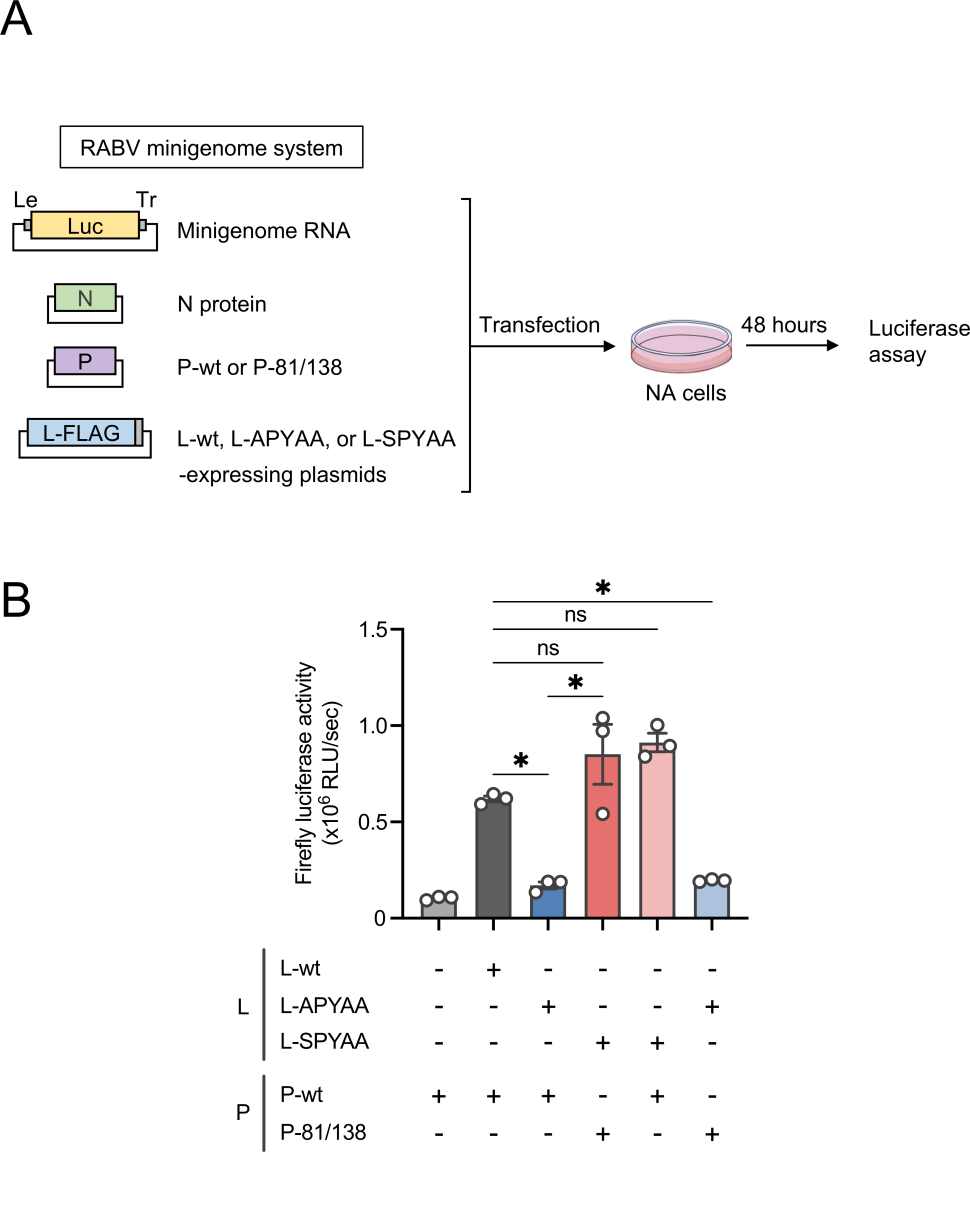
Compensatory effects of the RABV-APYAA*rev* P and L proteins on reduced RdRp function. (**A**) Schematic representation of the minigenome assay. NA cells were transfected with plasmids expressing the P and L proteins of RABV-wt, RABV-APYAA, and RABV-APYAA*rev* in various combinations together with plasmids expressing the N protein and minigenome RNA. As a negative control, an empty vector was transfected instead of the plasmid expressing the L protein. At 48 h.p.t., the transfected cells were lysed for a luciferase assay. Le and Tr indicate 3′- and 5′-terminal non-coding regions of the RABV-wt genome, respectively. (**B**) The minigenome assay was carried out in triplicate and repeated three times independently. The values in the graph are means ± SE of the means. Each dot represents the values obtained from each experiment. *, significant difference (*P* < 0.05); ns, not significant; RLU, relative light units.

Considering that the L protein interacts with the P protein to exert its RdRp function, the sequence analysis of RABV-APYAA*rev* suggests that one or some of the mutations found at P81/138 and L1929 compensate for the functional defect of the L protein that is caused by the APYAA substitution. Therefore, we examined the compensatory effects of these mutations by using a luciferase-based minigenome assay (Fig. 4A), which enables evaluation of the RdRp function based on the luciferase activity expressed from artificial minigenome RNA as a result of its replication/transcription driven by co-expression of the L and P proteins and the N protein.

In the minigenome assay, the coexpression of the L and P proteins from RABV-APYAA (L-APYAA and P-wt, respectively) generated a significantly lower luciferase activity than did coexpression of the L and P proteins from RABV-wt (L-wt and P-wt) (Fig. 4B). When the L and P proteins from RABV-APYAA*rev* (L-SPYAA and P-81/138) were coexpressed, the luciferase activity reached a level comparable to the activity produced by L-wt and P-wt, indicating that one or some of the mutations at P81/138 and L1929 compensate for the RdRp functional defect caused by the APYAA substitution. Importantly, the coexpression of L-SPYAA and P-wt, but not that of L-APYAA and P-81/138, yielded luciferase activity similar to the activity produced by L-wt and P-wt. These results indicate that the Ala-to-Ser mutation at L1929 is responsible for the compensation of the RdRp functional defect.

**Fig 5:**
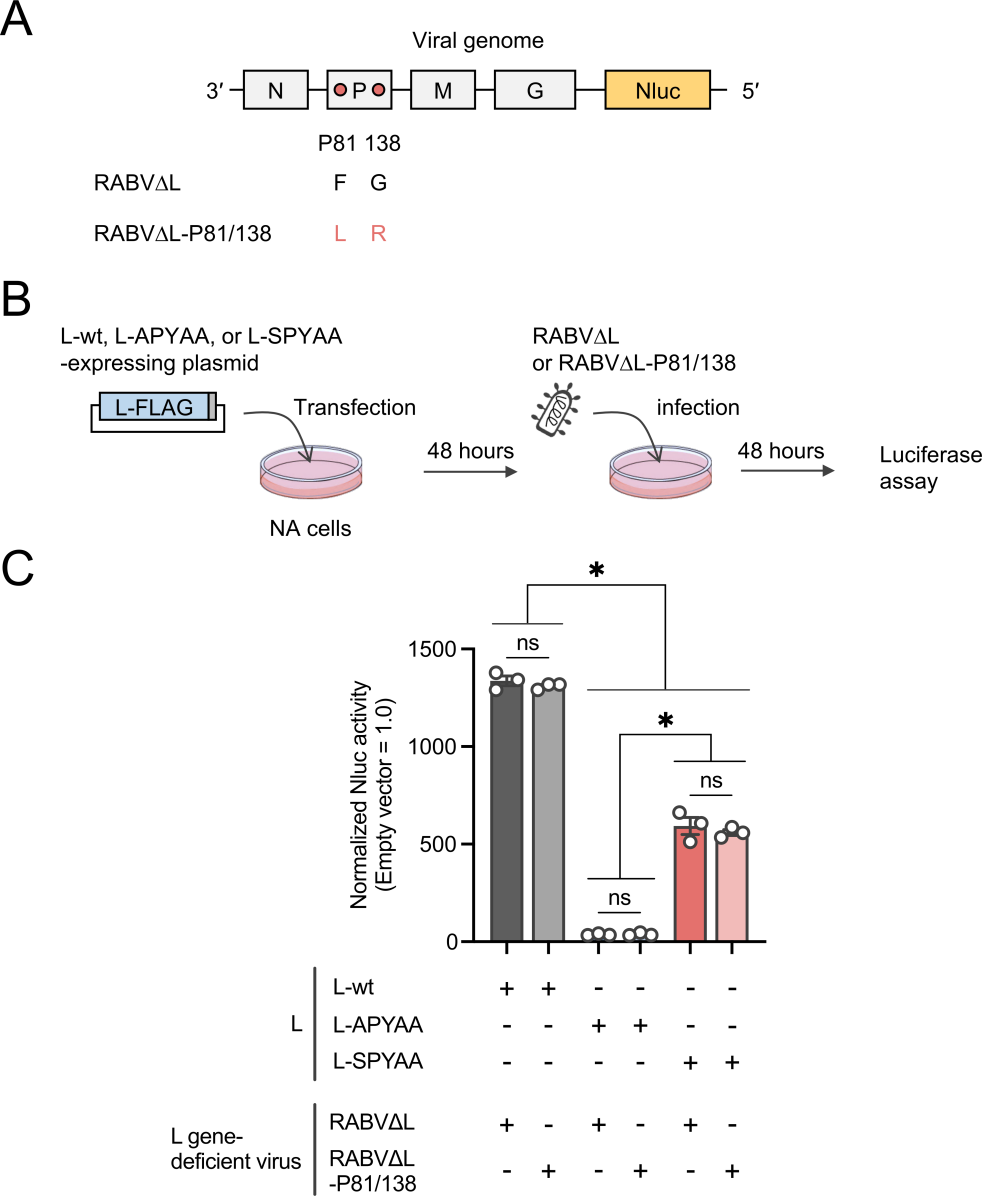
Importance of the Ala-to-Ser mutation at L1929 in functional compensation. (**A**) Schematic diagrams of the genome organization of RABV∆L and RABV∆L-P81/138. Gray boxes and a yellow box represent open reading frames derived from RABV-wt and an Nluc gene, respectively. Pink circles indicate amino acid residues that differ between RABV∆L and RABV∆L-P81/138. (**B**) Schematic representation of the *trans*-complementation assay using L gene-deficient viruses. NA cells were transfected with a plasmid expressing L-wt, L-APYAA, or L-SPYAA. As a negative control, an empty vector was transfected instead of the plasmid expressing the L protein. At 48 h.p.t., the transfected cells were infected with RABV∆L or RABV∆L-P81/138 at a multiplicity of infection of 0.01. After 48 hours, the infected cells were lysed to perform a luciferase assay. (**C**) The Nluc activities produced by each L gene-deficient virus in L protein-expressing cells are shown as ratios, considering the normalized Nluc activities produced in the cells transfected with an empty vector as 1.0. The assay was carried out in triplicate and repeated three times independently. The values in the graph are means ± SE of the means. Each dot represents the values obtained from a single experiment. *, significant difference (*P* < 0.05); ns, not significant.

Next, we aimed to validate the importance of an Ala-to-Ser mutation at L1929 in the functional compensation by using another experimental system, a *trans-*complementation assay system, that uses an L gene-deficient RABV (RABV∆L) and a plasmid expressing an L protein mutant in combination (29). In this system, the RABV∆L replicates and expresses NanoLuc luciferase (Nluc) under the support of the L protein mutant expressed from a plasmid, enabling evaluation of the RdRp function of the mutant based on the Nluc activity (Fig. 5A and B). To evaluate the compensatory effect of the mutations at P81/138 in addition to the mutation at L1929 by this assay, we newly generated an RABV∆L having Phe-to-Leu and Gly-to-Arg mutations at the respective positions in the P protein (RABV∆L-P81/138) (Fig. 5A). First, we confirmed that with support by L-wt, both RABV∆L and RABV∆L-P81/138 produced high levels of Nluc activity (Fig. 5C). We found that, compared to L-wt, L-APYAA assisted RABV∆L and RABV∆L-P81/138 less efficiently in expressing Nluc activity. Notably, L-SPYAA yielded significantly higher levels of Nluc activity from the respective L gene-deficient viruses than did L-APYAA. These findings confirmed that the Ala-to-Ser mutation at L1929, not the Phe-to-Leu and Gly-to-Arg mutations at P81/138, compensates for the RdRp functional defect caused by the APYAA substitution.

### Examination of compensatory effects of the mutations at P81/138 and L1929 on the reduced L-P interaction

**Fig 6:**
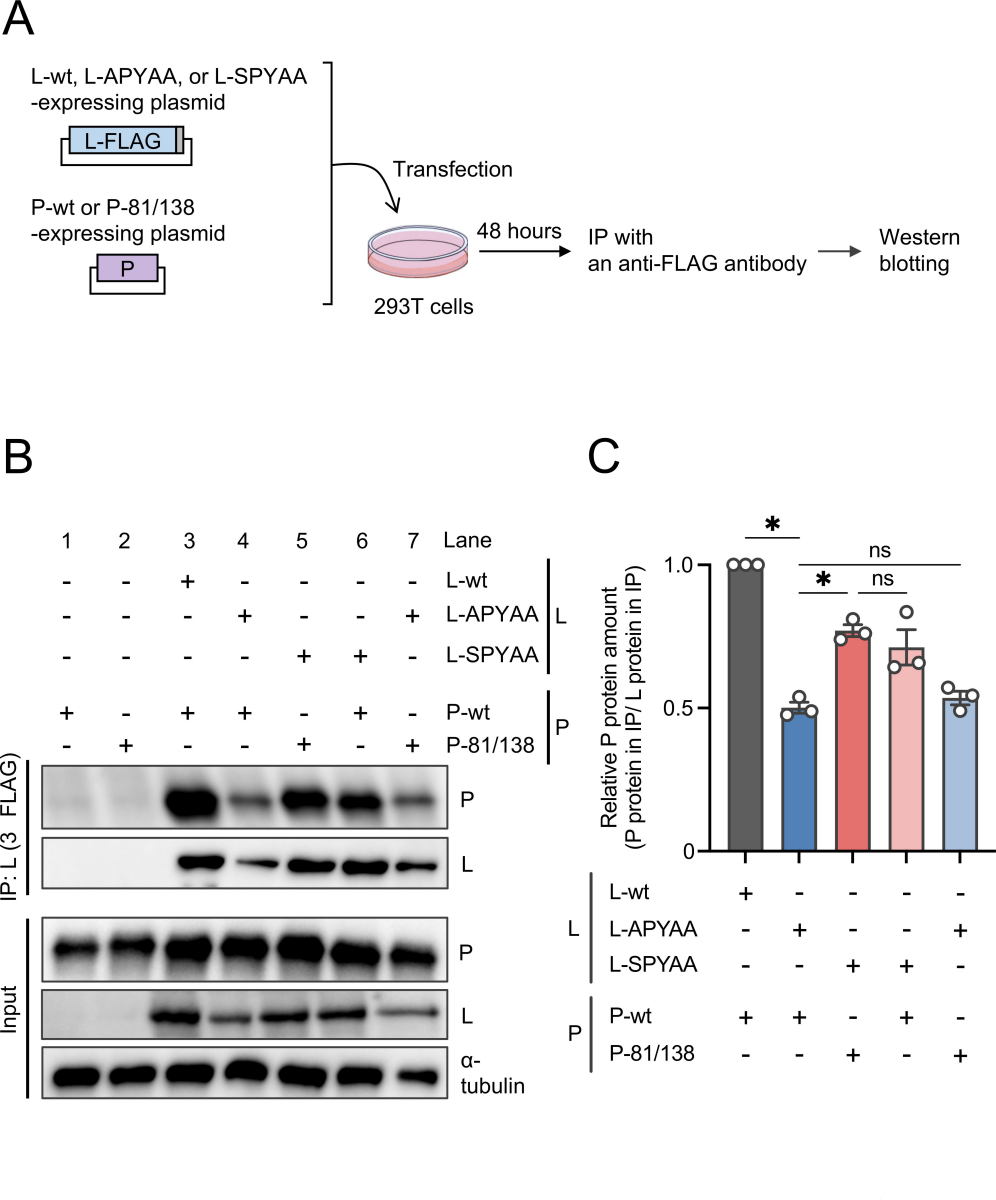
Compensatory effect of the Ala-to-Ser mutation at L1929 on reduced L-P interaction. (**A**) Schematic diagram of the Co-IP assay. 293T cells were transfected with a plasmid expressing P-wt or P-81/138 together with a plasmid expressing L-wt, L-APYAA, or L-SPYAA. As a negative control, an empty vector was transfected instead of the plasmid expressing the L protein. At 48 h.p.t., the transfected cells were lysed for immunoprecipitation with an anti-FLAG antibody to precipitate the L protein. The cell lysates and immunoprecipitates were subjected to Western blotting. (**B**) The P and L proteins in the cell lysates and immunoprecipitates were detected by Western blotting with an anti-P protein rabbit serum and anti-FLAG mouse monoclonal antibody, respectively. As a loading control, α-tubulin was also detected in the lysates by Western blotting with an anti-α-tubulin mouse monoclonal antibody. (**C**) The band intensities of the P and L proteins detected in the immunoprecipitates (panel B, IP) were quantified, and relative amounts of the precipitated P proteins to the precipitated L proteins are shown as ratios, considering the relative amounts of P-wt precipitated by L-wt as 1.0. The assay was carried out in triplicate and repeated three times independently. Western blotting shows a representative result of three independent replicates. The values in the graph are means ± SE of the means. Each dot represents the values obtained from a single experiment. *, significant difference (*P* < 0.05); ns, not significant.

Next, to examine whether the Ala-to-Ser mutation at L1929 compensated for the L-P interaction reduced by the APYAA substitution (29), we performed a coimmunoprecipitation (Co-IP) assay using 293T cells transfected to express the P and L proteins from RABV-wt, RABV-APYAA, and RABV-APYAA*rev* in various combinations (Fig. 6A). In this assay, we precipitated the L protein using an anti-FLAG tag antibody and detected the P protein in the immunoprecipitate. Since there was a variation in the amounts of L proteins in the immunoprecipitates (Fig. 6B, IP) probably due to a variation in expression levels of the L proteins in lysates of the transfected cells (Fig. 6B, input), we decided to determine the relative amounts of the P proteins to the L proteins in the respective precipitate samples by quantifying the band intensities of these proteins. The quantitative data indicated that, consistent with the findings in our previous study (29), Co-IP of L-wt resulted in the detection of a substantial amount of P-wt, whereas Co-IP of L-APYAA led to the detection of a significantly smaller amount of P-wt (Fig. 6C). These findings confirm that the APYAA substitution at L1929–1933 reduces the L-P interaction. We found that the Co-IP of L-SPYAA with P-81/138 yielded a larger amount of the P protein in the precipitate than did the Co-IP of L-APYAA with P-wt. Importantly, the Co-IP of L-SPYAA with P-wt, but not Co-IP of L-APYAA with P-81/138, resulted in the detection of the P protein with efficiency similar to that of Co-IP of L-SPYAA with P-81/138. These findings indicate that the Ala-to-Ser mutation at L1929 compensates for the L-P interaction reduced by the APYAA substitution.

### Functional importance of the three hydrophilic residues in the NPYNE sequence

**Fig 7:**
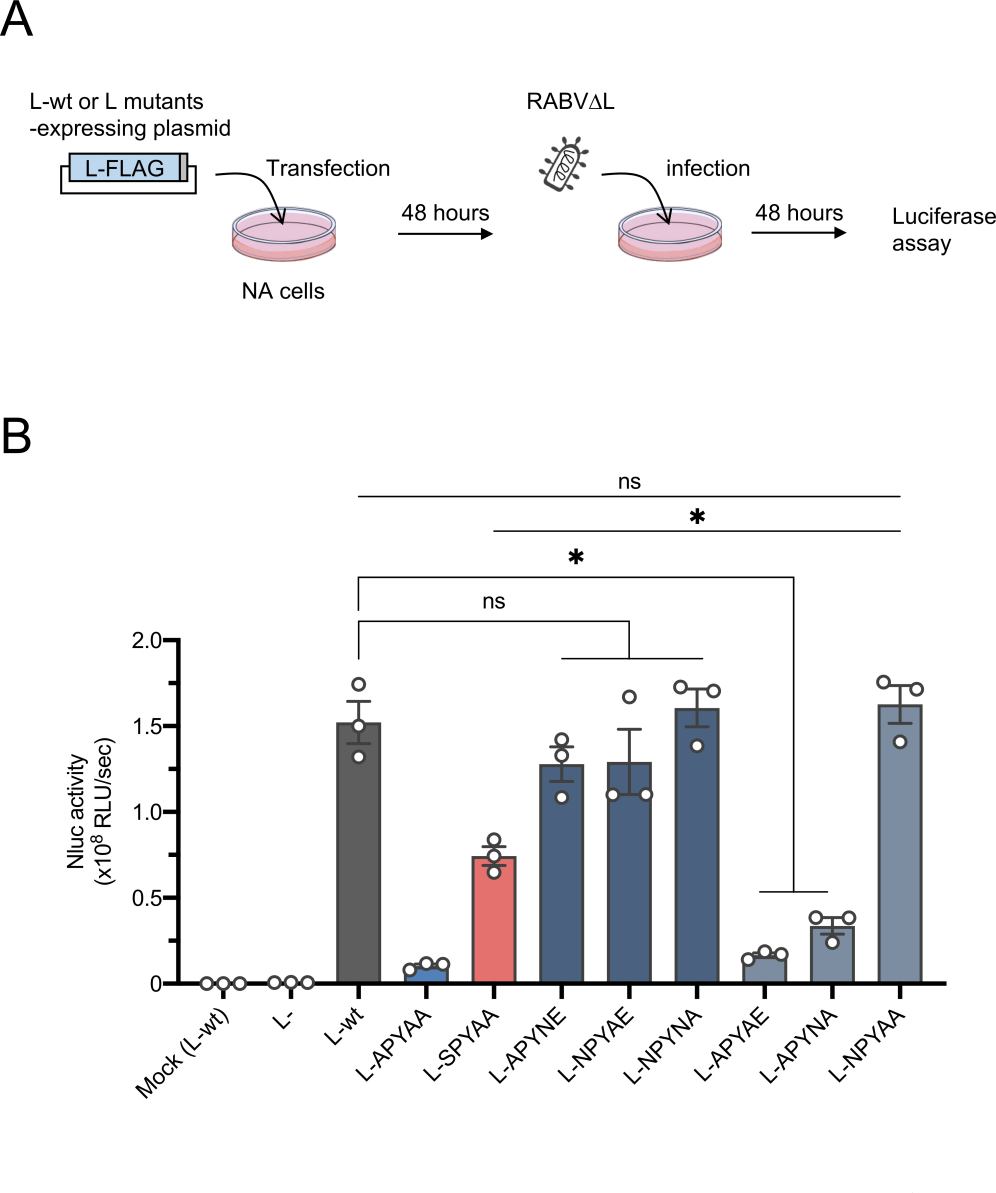
Importance of the three hydrophilic residues in the NPYNE sequence for RdRp function. (**A**) Schematic representation of the *trans*-complementation assay to examine the functional roles of the three hydrophilic residues of the NPYNE sequence. NA cells were transfected with a plasmid expressing L-wt, L-APYAA, L-SPYAA, or each of the L protein mutants harboring the Ala residue(s) instead of one or two of the three hydrophilic residues in the NPYNY sequence. As a negative control, an empty vector was transfected instead of the plasmid expressing the L protein. At 48 h.p.t., the transfected cells were infected with RABV∆L at a multiplicity of infection of 0.01. After 48 hours, the infected cells were lysed to perform a luciferase assay. (**B**) The *trans*-complementation assay was carried out in triplicate and repeated three times independently. The values in the graph are means ± SE of the means. Each dot represents the values obtained from a single experiment. *, significant difference (*P* < 0.05); ns, not significant; Mock (L-wt), cells expressing L-wt but not infected with RABV∆L; L-, cells transfected with an empty vector.

The findings described above strongly suggest that the first residue (Asn) of the NPYNE sequence at L1929–1933 is important for both the RdRp function and the P protein binding ability of the L protein. To investigate the functional importance of each hydrophilic residue in the NPYNE sequence at L1929–1933, including Asn at L1929, we constructed a series of plasmids expressing the L protein mutants, which had Ala residues instead of one or two of the three hydrophilic residues in the NPYNE sequence, and examined the RdRp function of each mutant by using the *trans*-complementation assay with RABV∆L (Fig. 7A).

All of the L protein mutants harboring one Ala substitution (L-APYNE, L-NPYAE, and L-NPYNA) assisted RABV∆L to produce similar levels of Nluc activities as the L-wt did (Fig. 7B), indicating that retention of two of the three hydrophilic residues is sufficient to maintain the RdRp function. Compared to L-wt, the mutants that retained only Asn at position 1932 in the L protein (L1932) or Glu at position 1933 in the L protein (L1933) (L-APYNA or L-APYAE, respectively) showed significantly lower Nluc activity. However, the mutant that retained only Asn at L1929 (L-NPYAA) yielded Nluc activity comparable to the activity produced by L-wt. These results indicate that Asn at L1929 plays the most important role in RdRp function among the hydrophilic residues in the NPYNE sequence.

### Importance of each hydrophilic residue in the NPYNE sequence in the L-P interaction

**Fig 8:**
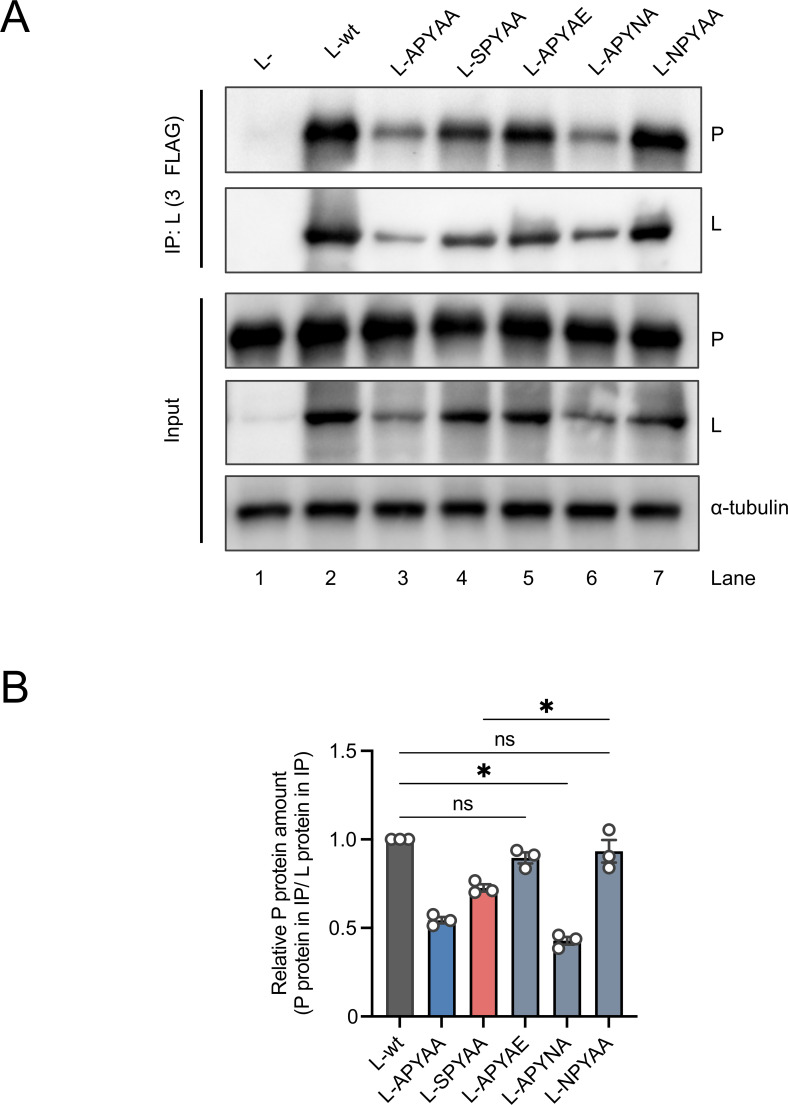
Importance of each hydrophilic residue in the NPYNE sequence for P protein binding. (**A**) Co-IP of the L proteins with P proteins. 293T cells were transfected with a plasmid expressing P-wt together with a plasmid expressing L-wt, L-APYAA, L-SPYAA, or each of the L protein mutants harboring two Ala substitutions in the NPYNE sequence. As a negative control, an empty vector was transfected instead of the plasmid expressing the L protein. At 48 h.p.t., the transfected cells were lysed for immunoprecipitation with an anti-FLAG antibody to precipitate the L protein. The lysates and immunoprecipitates were subjected to Western blotting with an anti-P protein rabbit serum, anti-FLAG mouse monoclonal antibody, and anti-α-tubulin mouse monoclonal antibody to detect the P protein, L protein, and α-tubulin, respectively. (**B**) The band intensities of the P and L proteins detected in the immunoprecipitates (panel A) were quantified, and relative amounts of the precipitated P proteins to the precipitated L proteins are shown as ratios, considering the relative amounts of P-wt precipitated by L-wt as 1.0. The assay was carried out in triplicate and repeated three times independently. Western blotting shows a representative result of three independent replicates. The values in the graph are means ± SE of the means. Each dot represents the values obtained from a single experiment. *, significant difference (*P* < 0.05); ns, not significant.

Next, we examined the importance of each hydrophilic residue in the NPYNE sequence in the L-P interaction by using a Co-IP assay. Since the amounts of L-APYAA and L-APYNA were remarkably smaller than that of L-wt in the immunoprecipitates (Fig. 8A, IP), we compared the relative amounts of the P proteins. L-wt, L-APYAE, and L-NPYAA were precipitated with substantial and comparable amounts of P-wt, whereas L-APYAA and L-NPYNA were precipitated with smaller amounts of P-wt (Fig. 8B). These results indicate that both Asn at L1929 and Glu at L1933 play an important role in the L-P interaction. It is notable that L-APYAE is competent in P protein binding ability but incompetent in RdRp function, while L-APYAA is incompetent in both functions (Fig. 7B and 8B). These results indicate that the L-P interaction is necessary but not sufficient for the L protein’s RdRp function.

It should also be noted here that, unlike RdRp-defective L-APYAE, L-APYNE is competent in RdRp function (Fig. 7B), indicating that in combination with Glu at L1933, which is critical for P protein binding (Fig. 8B), Asn at L1932 plays an important role in RdRp function.

### Stabilization of the L protein by binding with the P protein

**Fig 9:**
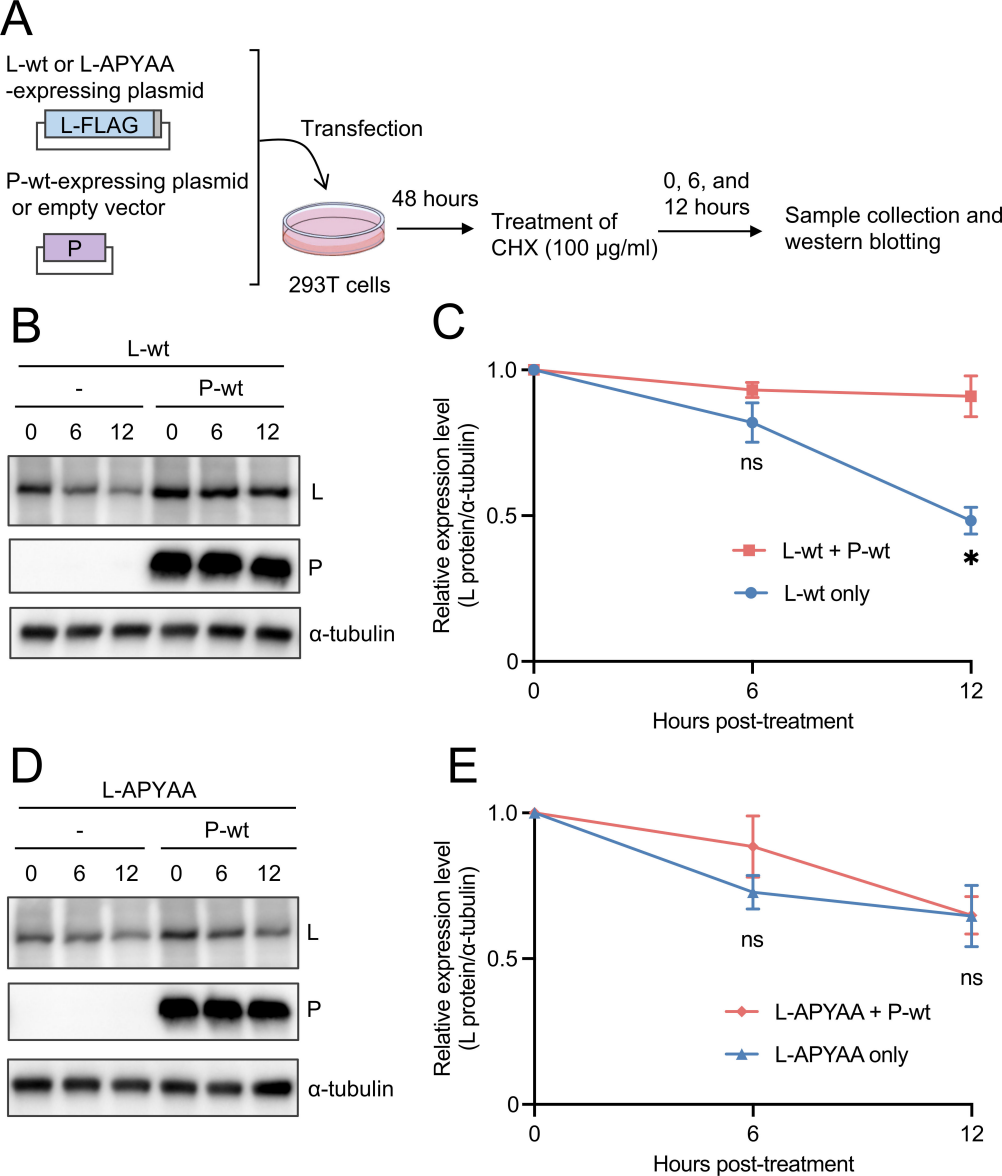
Stabilizing effect of the RABV P protein on L protein expression. (**A**) Schematic diagram of the CHX assay. 293T cells were transfected with a plasmid expressing L-wt or L-APYAA together with or without a plasmid expressing P-wt. At 48 h.p.t., the transfected cells were treated with 100 µg/ml of CHX and then harvested at 0, 6, and 12 hours after treatment for Western blotting. (**B and D**) The P protein, L protein, and α-tubulin in the cell lysates were detected by Western blotting with anti-P protein rabbit serum, anti-FLAG mouse monoclonal antibody, and anti-α-tubulin mouse monoclonal antibody, respectively. (**C and E**) The band intensities of the L protein and α-tubulin in each lysate were quantified, and relative expression levels of the L protein to α-tubulin are shown as ratios, considering the relative expression level at 0 hours post-treatment as 1.0. These assays were carried out in triplicate and repeated three times independently. Western blotting shows a representative result of three independent replicates. The values in the graph are means ± SE of the means. *, significant difference (*P* < 0.05); ns, not significant.

Based on the strong correlation between the P protein binding ability of the L protein mutants (Fig. 6C and 8B) and their expression levels in cell lysates prepared for the Co-IP (Fig. 6B and 8A, input), it is possible that the RABV P protein can stabilize L protein expression through the L-P interaction, as previously reported for the VSV P protein (13). To examine this possibility, we conducted a cycloheximide (CHX) chase assay (Fig. 9A). Specifically, 293T cells transfected to express L-wt or L-APYAA with or without the P protein were treated with a translation inhibitor, CHX, for 0, 6, and 12 hours. Relative expression levels of the L proteins to α-tubulins were determined by quantifying their band intensities in the respective lysates of the transfected cells and were shown as the ratio, considering the relative expression level at 0 hours post-treatment as 1.0.

When L-wt was expressed alone, its expression levels decreased over time, falling to approximately 50% at 12 hours post-treatment (Fig. 9B and C). By contrast, the coexpression of P-wt completely maintained the expression level of L-wt (Fig. 9B and C). The expression level of L-APYAA, which had reduced P protein-binding ability (29) (Fig. 6C and 8B), was decreased regardless of the coexpression of P-wt (Fig. 9D and E). These results indicate that the RABV L protein is stabilized by binding to the RABV P protein and that the NPYNE sequence at L1929–1933 plays an important role in this stabilization.

## DISCUSSION

The concept that the rhabdovirus L protein requires physical interaction with the P protein to exert its RdRp function and thus drive viral replication is widely accepted. However, little evidence supporting this concept has been obtained under an actual infection condition. The main reason is that disruption of the L-P interaction severely affects viral viability and therefore disables artificially generating and characterizing a virus mutant that loses the normal interaction. Surprisingly, in this study, we successfully established a RABV mutant, RABV-APYAA, in which the NPYNE sequence at L1929–1933 was substituted with APYAA that reduces the L-P interaction and also the L protein’s RdRp function (29). Our previous study demonstrated that the APYAA substitution did not completely destroy the L-P interaction (29), suggesting that the remaining interaction in RABV-APYAA was sufficient to maintain the RdRp function minimally required for viral viability. Through characterization of RABV-APYAA, we showed that the APYAA substitution remarkably reduces viral replication capacity and virulence (Fig. 2B and C and Fig. 3). These findings provide substantial evidence that the L-P interaction is important for RABV replication and pathogenesis, indicating a high potential of the L-P interaction as a target for anti-rabies therapeutics development.

In this study, we obtained and genetically analyzed a revertant of RABV-APYAA, RABV-APYAA*rev*, originally aiming to identify a mutation(s) in the P protein that could compensate for the reductions in L protein’s RdRp function and P protein-binding ability caused by the APYAA substitution at L1929–1933. Although the genetic analysis revealed that RABV-APYAA*rev* indeed has mutations in the P protein, at P81/138 (Table 1; Fig. 2A), our findings demonstrated that these mutations in the P protein do not compensate either the reduction in RdRp function or reduction in P protein-binding ability of the L protein (Fig. 4B, 5C, and 6C). Given that the mutations at P81/138 are located in a region that is not highly conserved among various lyssavirus species (33) (Fig. S2), it is reasonable that the region does not have an important role in interaction with the L protein. Meanwhile, our findings clearly indicate that the Ala-to-Ser mutation at L1929 compensates for the reductions in RdRp function and P protein-binding ability (Fig. 4B, 5C, and 6C), suggesting the importance of the first residue (Asn) in the NPYNE sequence at L1929–1933 in these functions. In fact, we demonstrated that Asn at L1929 plays an important role in both the RdRp function and the P protein binding (Fig. 7B and 8B).

In addition, the genetic analysis revealed that RABV-APYAA*rev* has a His-to-Asn mutation at position 21 in the G protein (Table 1; Fig. 2A). Since there is no report showing that the RABV G protein physically interacts with the L protein and/or the P protein, we consider that this mutation in the G protein does not affect L protein function. Meanwhile, we speculate that this mutation can contribute to the efficient growth of RABV-APYAA*rev* in NA cells (Fig. 2C). It will be interesting to check in the future whether the mutation functions to compensate for expression of the G protein reduced by the functional defect of the L protein.

In the published structure of the L-P_1–91_ complex (14), the functionally important Asn at L1929 is located at the very N-terminus of the CTD α1, the first α-helix that constitutes the CTD of the RABV L protein (Fig. 1B). In general, an Asn residue at the N-terminus of the α-helix forms a helix capping, a specific pattern of hydrogen bonds (34), which stabilizes the first turn of the α-helix structure (35). Therefore, there is the possibility that Asn at L1929 forms the helix capping and thus plays an important role in the formation and stabilization of the CTD α1. To clarify whether this is the case, it is necessary to determine a higher-resolution structure of the RABV L-P complex.

The findings obtained in this study demonstrate that hydrophilic residues other than Asn at L1929 in the NPYNE sequence also have important roles in the L protein functions. Specifically, characterization of the mutant L-APYAE revealed that Glu at L1933 is important for the L-P interaction (Fig. 7B and 8B). A comparison of the mutants L-APYAE and L-APYNE revealed that Asn at L1932 is critical for the RdRp function (Fig. 7B and 8B). Taken together, we conclude that each of the three hydrophilic residues in the NPYNE sequence at L1929–1933 has an important role in the L protein’s P protein-binding ability and/or RdRp function. Namely, Asn at L1929 is important for both functions, while Asn at L1932 and Glu at L1933 are pivotal for the RdRp function and the L-P interaction, respectively.

Despite the fact that the CTD α1 does not form an interface for P protein binding (Fig. 1B), our data highlight the importance of the CTD α1 in the L-P interaction. Although the underlying mechanism remains to be elucidated, structural rearrangement of the L protein might be involved in the L-P interaction regulated by the CTD α1. Several reports demonstrated that once the P protein binds, the positions of the CD, MT, and CTD are stabilized, and then the L protein structure is rearranged to a compact state (14, 28, 36–38). Based on these findings, we speculate that this L protein compaction induced by the P protein binding could increase the binding affinity between the L and P proteins. Since the CTD α1 is adjacent to the connector region between the MT and CTD, which is important for the structural rearrangement of the L protein (14, 28, 36, 38), it seems reasonable that this helix could be involved in the stabilization of the L-P interaction through the L protein compaction process.

Through functional analyses of various L protein mutants including L-APYAE, we showed for the first time that the RABV L-P interaction is necessary but not sufficient for the RdRp function (Fig. 7B and 8B). This finding clearly indicates that the structural requirement of the L protein for its RdRp function is inconsistent with that for binding with the P protein, providing a novel insight into the molecular function and structure of the L protein. Although the mechanism by which the CTD α1 structurally contributes to the RdRp function after the P protein binding remains unclear, we believe that functionally defective L protein mutants, such as L-APYAA and L-APYAE, will be useful for elucidating the mechanism by examining the molecular structure and dynamics of these mutants.

Although it was previously reported that L protein expression by VSV and other viruses within the order *Mononegavirales* is stabilized by binding with its P protein (13, 39–43), there has been no study in which it was rigorously examined whether the RABV L-P interaction stabilizes L protein expression. Data obtained in this study demonstrate that the RABV L protein is stabilized by binding with the P protein (Fig. 9B and C). Moreover, we showed that the NPYNE sequence has an important role in this stabilization (Fig. 9D and E). These findings suggest that the CTD α1, including the NPYNE sequence, is involved in this stabilization through its indirect contribution to the L-P interaction. Although the mechanism of the RABV L protein stabilization by the P protein binding remains unclear, the fact that the L-P interaction in other mononegaviruses protects their L protein from degradation via a cellular proteasome pathway (42–44) implies that the same mechanism can be applied to the RABV L-P interaction.

In conclusion, we demonstrate here the importance of the CTD α1 of the RABV L protein in its P protein binding, RdRp function, and stable expression. This is the first evidence indicating that the CTD region that does not form an interface with the P protein contributes to the rhabdovirus L-P interaction. Similar to the RABV L protein CTD α1, the first α-helix in the VSV L protein CTD is adjacent to the connector region between the MT and CTD (14, 15, 28). This structural similarity suggests that the indirect role of the CTD in the L-P interactions is conserved among various rhabdoviruses including RABV, VSV, and CHPV, which is an emerging viral pathogen genetically related to VSV (45). We believe that this study provides novel insights into the molecular structure, dynamics, and function of the L proteins of rhabdoviruses and thus useful information for developing antiviral therapeutics targeting the L-P interaction.

## MATERIALS AND METHODS

### Cells

Mouse neuroblastoma-derived NA cells (46) were maintained in E-MEM (Wako, Osaka, Japan) with 10% fetal calf serum (FCS) and 1% penicillin and streptomycin (PS) (Nacalai Tesque, Kyoto, Japan). Human embryonic kidney-derived HEK293T cells (ATCC: CRL3216) were grown in D-MEM (Thermo Fisher Scientific, Waltham, MA, USA) with 10% FCS, 1% PS, and 0.5% amphotericin B (Sigma-Aldrich, St. Louis, MO, USA). TetOff-NiL-NA cells, an NA cell clone constitutively expressing L-wt, were cultured as reported previously (29). BHK/T7-9 cells (47), a BHK cell clone constitutively expressing T7 polymerase, were maintained in E-MEM (Wako) with 5% FCS, 1% PS, and 10% triphosphate broth (Becton Dickinson, Franklin Lakes, NJ, USA).

### Viruses

RABV-wt (recombinant Nishigahara strain) was recovered from its full-length genome plasmid as previously reported (32). The RABV-wt mutant RABV-APYAA, in which amino acids at L1929, L1932, and L1933 were substituted with Ala residues, was generated by the reverse genetics system of the Nishigahara strain (32). RABV-APYAA*rev*, the revertant virus of RABV-APYAA, was established by serial passages in NA cells as described below. Stocks of these viruses were prepared in suspensions of NA cells and stored at −80°C until use. RABV∆L, RABV-wt with the Nluc gene instead of the L gene, was prepared as in a previous study (29). RABV∆L-P81/138 was recovered from its full-length genome plasmid by using the reverse genetics system of the Nishigahara strain (32). These L-gene-deficient virus stocks were prepared in TetOff-NiL-NA cells and stored at −80°C until use.

### Plasmids

A plasmid expressing the C-terminally 3×FLAG-tagged L-wt (pCNiL-3×FLAG) in a mammalian expression vector, pCAGGS/MCS (48), was generated in a previous study (29). Plasmids expressing the C-terminally 3×FLAG-tagged L-APYAA or L-SPYAA (pCNiL-APYAA-3×FLAG or pCNiL-SPYAA-3×FLAG, respectively) were constructed by substituting the NPYNE sequence at positions 1929–1933 of the L protein in pCNiL-3×FLAG with the APYAA or SPYAA sequence, respectively. Similarly, plasmids expressing respective C-terminally 3×FLAG-tagged L protein mutants, in which one or two hydrophilic residues in the NPYNE sequence were substituted with Ala residues, were constructed (pCNiL-APYNE-3×FLAG, pCNiL-NPYAE-3×FLAG, pCNiL-NPYNA-3×FLAG, pCNiL-APYAE-3×FLAG, pCNiL-APYNA-3×FLAG, and pCNiL-NPYAA-3×FLAG, respectively [substituted residues are underlined]). Plasmids expressing the RABV-wt N protein or P-wt in pCAGGS/MCS (pCAGGS-NiN or pCAGGS-NiP, respectively) were generated in a previous study (49). A plasmid expressing P-81/138, pCAGGS-NiP-P81/138, was constructed by replacing the P gene of RABV-wt in pCAGGS-NiP with that of RABV-APYAA*rev*. A minigenome plasmid, pC-NiDI-Luc, which expresses a negative-sense minigenome RNA encoding firefly luciferase, was constructed in a previous study (29).

The full-length genome plasmid of RABV-APYAA was constructed by substituting amino acid residues at L1929, L1932, and L1933 of the full-length genome plasmid of RABV-wt (32) with Ala residues. The full-length genome plasmid of RABV∆L-P81/138 was constructed by substituting amino acid residues at P81/138 of the full-length genome plasmid of RABV∆L with Leu and Arg residues, respectively.

Details on the construction methods and sequences of plasmids are available on request.

### Serial passages

The undiluted stock of RABV-APYAA was inoculated into monolayered NA cells cultured in a 96-well plate. After 4 days, the culture medium was collected and centrifuged at 1,500 × *g* for 10 minutes. The supernatant was used to infect new monolayered NA cells. This procedure was repeated 20 times to establish the passaged virus.

### Cloning by limited dilution

NA cells (2.0 × 10^4^ cells/well) were cultured in 96-well plates for 2 days before virus infection. The cells were infected with 25 µL/well of the twofold diluted passaged virus. The culture medium was harvested at 7 days post-infection (d.p.i.) and stored at −80°C until use. The remaining cells were immunostained with an anti-RABV N protein monoclonal antibody (50) as described previously (51) to confirm the antigen-positive rate. The culture medium from an antigen-positive well in a dilution series with an antigen-positive rate of less than 5% was inoculated into NA cell monolayers (1.0 × 10^5^ cells/well) that were grown in a 24-well plate for 2 days. At 4 d.p.i., the culture medium was collected and centrifuged at 1,500 × *g* for 10 minutes to establish the cloned virus, RABV-APYAA*rev*.

### Focus formation

NA cells (6.0 × 10^4^ cells/well) that were cultured for 2 days in an 8-well chamber slide were infected with RABV-wt, RABV-APYAA, or RABV-APYAA*rev* at a multiplicity of infection (MOI) of 0.01 and were then overlaid with E-MEM containing 5% FCS, 1% PS, 0.5% amphotericin B, and 1% methylcellulose 4000 (nacalai tesque). At 2 d.p.i., the infected cells were immunostained with the anti-RABV N protein monoclonal antibody (50) as described previously (51). Virus foci were observed by using the Biozero fluorescence microscope BZ-800 series (Keyence, Osaka, Japan).

### Growth curve

NA cells (1.0 × 10^6^ cells/bottle) that were cultured for 2 days in a T-25 flask were infected with RABV-wt, RABV-APYAA, or RABV-APYAA*rev* at an MOI of 0.01. The culture supernatant was harvested at 0, 1, 3, and 5 d.p.i., and the virus titer was titrated by a focus assay as reported previously (51).

### Virulence in mice

Five 6-week-old male ddY mice (Japan SLC Inc., Shizuoka, Japan) were inoculated intracerebrally with 0.03 mL of 10^3^ focus-forming units/mouse of RABV-wt, RABV-APYAA, or RABV-APYAA*rev*. The infected mice were observed daily for 14 days to record body weight changes and survival rates. Mice were euthanized when they lost their righting reflex.

### Next-generation sequencing

The nucleotide sequences of stocks of RABV-wt, RABV-APYAA, and RABV-APYAA*rev* were determined by using a next-generation sequencer as described previously (52).

### Minigenome assay

NA cells (1.5 × 10^5^ cells/well) were grown in a 24-well plate for 24 hours. The cells were then transfected with 0.06 µg of pCAGGS-NiP or pCAGGS-NiP-P81/138 and 0.4 µg of pCAGGS/MCS, pCNiL-3×FLAG, pCNiL-APYAA-3×FLAG, or pCNiL-SPYAA-3×FLAG together with 0.06 µg of pCAGGS-NiN and 0.4 µg of pC-NiDI-Luc using 3.9 µL of Lipofectamine 2000 Transfection Reagent (Invitrogen). At 48 hours post-transfection (h.p.t.), the cells were washed with phosphate-buffered saline (PBS) (-) (Nissui, Tokyo, Japan) and then lysed with 100 µL of Cell Culture Lysis Reagent (Promega, Madison, WI, USA). The cell lysates were centrifuged at 13,200 × *g* for 2 minutes at 4°C. The firefly luciferase activity in the supernatant was measured using a Luciferase Assay System (Promega) and GloMax 20/20 Luminometer (Promega).

### *trans*-Complementation assay

NA cells (1.5 × 10^5^ cells/well) that were grown in a 24-well plate for 24 hours were transfected with 0.5 µg of pCAGGS/MCS, pCNiL-3×FLAG, pCNiL-APYAA-3×FLAG, pCNiL-SPYAA-3×FLAG, pCNiL-APYNE-3×FLAG, pCNiL-NPYAE-3×FLAG, pCNiL-NPYNA-3×FLAG, pCNiL-APYAE-3×FLAG, pCNiL-APYNA-3×FLAG, or pCNiL-NPYAA-3×FLAG using 1.5 µL of TransIT-2020 Transfection Reagent (Mirus Bio LLC, Madison, WI, USA). After 48 hours, the cells were infected with RABV∆L or RABV∆L-P81/138 at an MOI of 0.01. At 2 d.p.i., the cells were washed with PBS (-) (Nissui) and then lysed with 100 µL of Passive Lysis Buffer (Promega). The cell lysates were collected and centrifuged at 13,200 × *g* for 2 minutes at 4°C. The Nluc activity in the supernatant was measured using the Nano-Glo Luciferase Assay System (Promega) and GloMax 20/20 Luminometer (Promega). To compare the Nluc activities produced by RABV∆L and RABV∆L-P81/138, Nluc activities produced by each L gene-deficient virus in L protein-expressing cells were normalized to those produced in the cells transfected with pCAGGS/MCS.

### Co-IP assay

293T cells (7.5 × 10^5^ cells/well) were grown in six-well plates coated with poly-D-lysine (Sigma-Aldrich) for 24 hours and were transfected with 2.5 µg of pCAGGS-NiP or pCAGGS-NiP-P81/138 and 7.5 µg of pCAGGS/MCS, pCNiL-3×FLAG, pCNiL-APYAA-3×FLAG, pCNiL-SPYAA-3×FLAG, pCNiL-APYNE-3×FLAG, pCNiL-NPYAE-3×FLAG, pCNiL-NPYNA-3×FLAG, pCNiL-APYAE-3×FLAG, pCNiL-APYNA-3×FLAG, or pCNiL-NPYAA-3×FLAG by using 30 µL of TransIT-X2 Dynamic Delivery System (Mirus Bio LLC). After 48 hours, the cells were washed with PBS (-) and lysed with RIPA buffer (50 mM Tris-HCl [pH 8.0], 150 mM NaCl, 1% NP-40, cOmplete mini protease inhibitor cocktail tablet [Roche, Basel, Switzerland]). The cell lysates were collected and centrifuged at 13,200 × *g* for 10 minutes at 4°C. Two hundred microliters of the supernatant was mixed with 200 µL of SureBeads Protein G magnetic beads (Bio-Rad, Hercules, CA, USA) previously washed with PBS (-) containing 0.1% Tween 20 (PBS-T) and then incubated with 2 µg of ANTI-FLAG M2 mouse monoclonal antibody (Sigma-Aldrich) for 2 hours at 4°C with rotation. The remaining supernatant was mixed with an equal volume of 2× Sample Buffer Solution (2ME+) (Wako) and incubated for 5 minutes at 95°C. After washing the beads three times with PBS-T, the beads were resuspended in 50 µL of RIPA buffer. To elute immunoprecipitants, the resuspended beads were incubated with an equal volume of 2× Sample Buffer Solution (2ME+) (Wako) for 5 minutes at 95°C, and the beads were removed by magnetization. Immunoprecipitates and supernatants of the cell lysates were analyzed by Western blotting as described below.

### Western blotting

Samples were separated by sodium dodecyl sulfate-polyacrylamide gel electrophoresis and transferred to a 0.45 µm polyvinylidene difluoride membrane Immobilon-P (Millipore, Billerica, MA, USA) using the Trans-Blot Turbo Transfer System (Bio-Rad). To separate the P protein and α-tubulin or L protein, 10% or 5% polyacrylamide gels were used, respectively. After blocking with PBS-T containing 5% nonfat dry milk for 60 minutes at room temperature, the bands of the FLAG-tagged L protein, P protein, and α-tubulin on the membrane were detected with ANTI-FLAG M2 mouse monoclonal antibody (:200, Sigma-Aldrich), anti-RABV P protein rabbit serum reported previously (:10,000) (29), and Monoclonal Anti-α-tubulin antibody produced in a mouse (:1,000, Sigma-Aldrich), respectively. HRP-conjugated anti-mouse IgG (:5,000, MP Biomedicals, Irvine, CA, USA) or HRP-conjugated anti-rabbit IgG (:100,000, MP Biomedicals) were used as secondary antibodies to detect the bands of L protein and α-tubulin or P protein, respectively. The bands were visualized using a Western Lightning Ultra (Perkin Elmer, Waltham, MA, USA) and detected by ChemiDoc XRS + System (Bio-Rad). The intensities of the bands were measured by Image Lab Software (ver.6.1.0, Bio-Rad).

### CHX chase assay

293T cells (1.5 × 10^5^ cells/well) were grown in 24-well plates coated with poly-D-lysine (Sigma-Aldrich) for 24 hours and were then transfected with 0.5 µg of pCAGGS/MCS or pCAGGS-NiP and 1.5 µg of pCNiL-3×FLAG or pCNiL-APYAA-3×FLAG by using 6 µL of the TransIT-X2 Dynamic Delivery System (Mirus Bio LLC). At 48 h.p.t., the culture medium was replaced with a medium containing 100 µg/mL of CHX (Wako). After 0, 6, and 12 hours of treatment with CHX, the cells were washed with PBS (-) and harvested using RIPA buffer. The cell lysates were centrifuged at 13,200 × *g* for 10 minutes at 4°C. Fifty microliters of the supernatant was mixed with an equal volume of sample buffer solution (2ME+) (Wako) and then boiled at 95°C for 5 minutes. Amounts of the P protein, L protein, and α-tubulin in the samples were analyzed by Western blotting as described above.

### Structural model

The structural model of the RABV (SAD-B19 strain) L protein in complex with P_1–91_ was downloaded from the Protein Data Bank (Protein Data Bank identifier [PDB ID]: 6UEB) and visualized by UCSF ChimeraX software (ver. 1.4) (53).

### Statistical analysis

For analysis of the replication capacity of the respective strain, two-way analysis of variance (ANOVA) with Tukey’s multiple comparison test was performed. Stabilities of L-wt and L-APYAA were analyzed using two-way ANOVA with Sidak’s multiple comparison test. For other statistical analyses, one-way ANOVA with Tukey’s multiple comparison test was conducted. All statistical analyses were performed by using GraphPad Prism 9 software (ver. 9.5.1, GraphPad Software, Boston, MA, USA). A *P* value of 0.05 or less was considered significant.

## Data Availability

Data supporting the findings in this study are available upon request from the corresponding author.

## SUPPLEMENTAL MATERIAL

Fig. S1 and S2 jvi.02082-24-s0001.pdf Alignments of amino acid sequences of lyssavirus L and P proteins.

ASM does not own the copyrights to Supplemental Material that may be linked to, or accessed through, an article. The authors have granted ASM a non-exclusive, world-wide license to publish the Supplemental Material files. Please contact the corresponding author directly for reuse.

## References

[1] Hampson K, Coudeville L, Lembo T, Sambo M, Kieffer A, Attlan M, Barrat J, Blanton JD, Briggs DJ, Cleaveland S, et al.. 2015. Global alliance for rabies control partners for rabies prevention. PLoS Negl Trop Dis 9:e0003709. doi:10.1371/journal.pntd.0003709 25881058 PMC4400070

[2] Rao BL, Basu A, Wairagkar NS, Gore MM, Arankalle VA, Thakare JP, Jadi RS, Rao KA, Mishra AC. 2004. A large outbreak of acute encephalitis with high fatality rate in children in Andhra Pradesh, India, in 2003, associated with Chandipura virus. Lancet 364:869–874. doi:10.1016/S0140-6736(04)16982-1 15351194 PMC7137741

[3] Menghani S, Chikhale R, Raval A, Wadibhasme P, Khedekar P. 2012. Chandipura virus: an emerging tropical pathogen. Acta Trop 124:1–14. doi:10.1016/j.actatropica.2012.06.001 22721825

[4] Chadha MS, Arankalle VA, Jadi RS, Joshi MV, Thakare JP, Mahadev PVM, Mishra AC. 2005. An outbreak of Chandipura virus encephalitis in the eastern districts of Gujarat state, India. Am J Trop Med Hyg 73:566–570. doi:10.4269/ajtmh.2005.73.566 16172482

[5] Gurav YK, Tandale BV, Jadi RS, Gunjikar RS, Tikute SS, Jamgaonkar AV, Khadse RK, Jalgaonkar SV, Arankalle VA, Mishra AC. 2010. Chandipura virus encephalitis outbreak among children in Nagpur division, Maharashtra, 2007. Indian J Med Res 132:395–399. 20966517

[6] Tandale BV, Tikute SS, Arankalle VA, Sathe PS, Joshi MV, Ranadive SN, Kanojia PC, Eshwarachary D, Kumarswamy M, Mishra AC. 2008. Chandipura virus: a major cause of acute encephalitis in children in North Telangana, Andhra Pradesh, India. J Med Virol 80:118–124. doi:10.1002/jmv.21041 18041027

[7] Devi S. 2024. India facing largest Chandipura virus outbreak in 20 years. Lancet 404:919. doi:10.1016/S0140-6736(24)01861-0 39245046

[8] Ogino T, Green TJ. 2019. RNA synthesis and capping by non-segmented negative strand RNA viral polymerases: lessons from a prototypic virus. Front Microbiol 10:1490. doi:10.3389/fmicb.2019.01490 31354644 PMC6636387

[9] Ogino M, Ito N, Sugiyama M, Ogino T. 2016. The Rabies virus L protein catalyzes mRNA capping with GDP polyribonucleotidyltransferase activity. Viruses 8:144. doi:10.3390/v8050144 27213429 PMC4885099

[10] Ogino T, Green TJ. 2019. Transcriptional control and mRNA capping by the GDP polyribonucleotidyltransferase domain of the rabies virus large protein. Viruses 11:504. doi:10.3390/v11060504 31159413 PMC6631705

[11] Ogino M, Gupta N, Green TJ, Ogino T. 2019. A dual-functional priming-capping loop of rhabdoviral RNA polymerases directs terminal de novo initiation and capping intermediate formation. Nucleic Acids Res 47:299–309. doi:10.1093/nar/gky1058 30395342 PMC6326812

[12] Holloway BP, Obijeski JF. 1980. Rabies virus-induced RNA synthesis in BHK21 cells. J Gen Virol 49:181–195. doi:10.1099/0022-1317-49-1-181 7420063

[13] Canter DM, Perrault J. 1996. Stabilization of vesicular stomatitis virus L polymerase protein by P protein binding: a small deletion in the C-terminal domain of L abrogates binding. Virology (Auckl) 219:376–386. doi:10.1006/viro.1996.0263 8638403

[14] Horwitz JA, Jenni S, Harrison SC, Whelan SPJ. 2020. Structure of a rabies virus polymerase complex from electron cryo-microscopy. Proc Natl Acad Sci U S A 117:2099–2107. doi:10.1073/pnas.1918809117 31953264 PMC6995008

[15] Jenni S, Bloyet L-M, Diaz-Avalos R, Liang B, Whelan SPJ, Grigorieff N, Harrison SC. 2020. Structure of the vesicular stomatitis virus L protein in complex with its phosphoprotein cofactor. Cell Rep 30:53–60. doi:10.1016/j.celrep.2019.12.024 31914397 PMC7049099

[16] Gilman MSA, Liu C, Fung A, Behera I, Jordan P, Rigaux P, Ysebaert N, Tcherniuk S, Sourimant J, Eléouët J-F, Sutto-Ortiz P, Decroly E, Roymans D, Jin Z, McLellan JS. 2019. Structure of the respiratory syncytial virus polymerase complex. Cell 179:193–204. doi:10.1016/j.cell.2019.08.014 31495574 PMC7111336

[17] Pan J, Qian X, Lattmann S, El Sahili A, Yeo TH, Jia H, Cressey T, Ludeke B, Noton S, Kalocsay M, Fearns R, Lescar J. 2020. Structure of the human metapneumovirus polymerase phosphoprotein complex. Nature 577:275–279. doi:10.1038/s41586-019-1759-1 31698413 PMC6949429

[18] Cao D, Gao Y, Roesler C, Rice S, D’Cunha P, Zhuang L, Slack J, Domke M, Antonova A, Romanelli S, Keating S, Forero G, Juneja P, Liang B. 2020. Cryo-EM structure of the respiratory syncytial virus RNA polymerase. Nat Commun 11:368. doi:10.1038/s41467-019-14246-3 31953395 PMC6969064

[19] Abdella R, Aggarwal M, Okura T, Lamb RA, He Y. 2020. Structure of a paramyxovirus polymerase complex reveals a unique methyltransferase-CTD conformation. Proc Natl Acad Sci U S A 117:4931–4941. doi:10.1073/pnas.1919837117 32075920 PMC7060699

[20] Yuan B, Peng Q, Cheng J, Wang M, Zhong J, Qi J, Gao GF, Shi Y. 2022. Structure of the Ebola virus polymerase complex. Nature 610:394–401. doi:10.1038/s41586-022-05271-2 36171293 PMC9517992

[21] Cong J, Feng X, Kang H, Fu W, Wang L, Wang C, Li X, Chen Y, Rao Z. 2023. Structure of the Newcastle Disease Virus L protein in complex with tetrameric phosphoprotein. Nat Commun 14:1324. doi:10.1038/s41467-023-37012-y 36898997 PMC10006412

[22] Peng Q, Yuan B, Cheng J, Wang M, Gao S, Bai S, Zhao X, Qi J, Gao GF, Shi Y. 2023. Molecular mechanism of de novo replication by the Ebola virus polymerase. Nature 622:603–610. doi:10.1038/s41586-023-06608-1 37699521

[23] Cao D, Gao Y, Chen Z, Gooneratne I, Roesler C, Mera C, D’Cunha P, Antonova A, Katta D, Romanelli S, Wang Q, Rice S, Lemons W, Ramanathan A, Liang B. 2024. Structures of the promoter-bound respiratory syncytial virus polymerase. Nature 625:611–617. doi:10.1038/s41586-023-06867-y 38123676 PMC10794133

[24] Xie J, Ouizougun-Oubari M, Wang L, Zhai G, Wu D, Lin Z, Wang M, Ludeke B, Yan X, Nilsson T, Gao L, Huang X, Fearns R, Chen S. 2024. Structural basis for dimerization of a paramyxovirus polymerase complex. Nat Commun 15:3163. doi:10.1038/s41467-024-47470-7 38605025 PMC11009304

[25] Li T, Liu M, Gu Z, Su X, Liu Y, Lin J, Zhang Y, Shen Q-T. 2024. Structures of the mumps virus polymerase complex via cryo-electron microscopy. Nat Commun 15:4189. doi:10.1038/s41467-024-48389-9 38760379 PMC11101452

[26] Yang G, Wang D, Liu B. 2024. Structure of the Nipah virus polymerase phosphoprotein complex. Nat Commun 15:8673. doi:10.1038/s41467-024-52701-y 39375338 PMC11458586

[27] Liang B. 2020. Structures of the Mononegavirales polymerases. J Virol 94:e00175-20. doi:10.1128/JVI.00175-20 32847861 PMC7592205

[28] Liang B, Li Z, Jenni S, Rahmeh AA, Morin BM, Grant T, Grigorieff N, Harrison SC, Whelan SPJ. 2015. Structure of the L protein of vesicular stomatitis virus from electron cryomicroscopy. Cell 162:314–327. doi:10.1016/j.cell.2015.06.018 26144317 PMC4557768

[29] Nakagawa K, Kobayashi Y, Ito N, Suzuki Y, Okada K, Makino M, Goto H, Takahashi T, Sugiyama M. 2017. Molecular function analysis of rabies virus RNA polymerase L protein by using an L gene-deficient virus. J Virol 91:e00826-17. doi:10.1128/JVI.00826-17 28768857 PMC5625484

[30] Chenik M, Schnell M, Conzelmann KK, Blondel D. 1998. Mapping the interacting domains between the rabies virus polymerase and phosphoprotein. J Virol 72:1925–1930. doi:10.1128/JVI.72.3.1925-1930.1998 9499045 PMC109484

[31] Castel G, Chtéoui M, Caignard G, Préhaud C, Méhouas S, Réal E, Jallet C, Jacob Y, Ruigrok RWH, Tordo N. 2009. Peptides that mimic the amino-terminal end of the rabies virus phosphoprotein have antiviral activity. J Virol 83:10808–10820. doi:10.1128/JVI.00977-09 19706704 PMC2753138

[32] Yamada K, Ito N, Takayama-Ito M, Sugiyama M, Minamoto N. 2006. Multigenic relation to the attenuation of rabies virus. Microbiol Immunol 50:25–32. doi:10.1111/j.1348-0421.2006.tb03767.x 16428870

[33] Gerard FCA, Ribeiro E de A Jr, Leyrat C, Ivanov I, Blondel D, Longhi S, Ruigrok RWH, Jamin M. 2009. Modular organization of rabies virus phosphoprotein. J Mol Biol 388:978–996. doi:10.1016/j.jmb.2009.03.061 19341745

[34] Aurora R, Rose GD. 1998. Helix capping. Protein Sci 7:21–38. doi:10.1002/pro.5560070103 9514257 PMC2143812

[35] Richardson JS, Richardson DC. 1988. Amino acid preferences for specific locations at the ends of alpha helices. Science 240:1648–1652. doi:10.1126/science.3381086 3381086

[36] Rahmeh AA, Schenk AD, Danek EI, Kranzusch PJ, Liang B, Walz T, Whelan SPJ. 2010. Molecular architecture of the vesicular stomatitis virus RNA polymerase. Proc Natl Acad Sci USA 107:20075–20080. doi:10.1073/pnas.1013559107 21041632 PMC2993402

[37] Gould JR, Qiu S, Shang Q, Dokland T, Ogino T, Petit CM, Green TJ. 2021. Consequences of phosphorylation in a Mononegavirales polymerase-cofactor system. J Virol 95:e02180-20. doi:10.1128/JVI.02180-20 33441337 PMC8092687

[38] Rahmeh AA, Morin B, Schenk AD, Liang B, Heinrich BS, Brusic V, Walz T, Whelan SPJ. 2012. Critical phosphoprotein elements that regulate polymerase architecture and function in vesicular stomatitis virus. Proc Natl Acad Sci U S A 109:14628–14633. doi:10.1073/pnas.1209147109 22908284 PMC3437890

[39] Smallwood S, Ryan KW, Moyer SA. 1994. Deletion analysis defines a carboxyl-proximal region of sendai virus P protein that binds to the polymerase L protein. Virology (Auckl) 202:154–163. doi:10.1006/viro.1994.1331 8009828

[40] Horikami SM, Smallwood S, Bankamp B, Moyer SA. 1994. An amino-proximal domain of the L protein binds to the P protein in the measles virus RNA polymerase complex. Virology (Auckl) 205:540–545. doi:10.1006/viro.1994.1676 7975255

[41] Sourimant J, Rameix-Welti M-A, Gaillard A-L, Chevret D, Galloux M, Gault E, Eléouët J-F. 2015. Fine mapping and characterization of the L-polymerase-binding domain of the respiratory syncytial virus phosphoprotein. J Virol 89:4421–4433. doi:10.1128/JVI.03619-14 25653447 PMC4442346

[42] Bloyet L-M, Welsch J, Enchery F, Mathieu C, de Breyne S, Horvat B, Grigorov B, Gerlier D. 2016. HSP90 chaperoning in addition to phosphoprotein required for folding but not for supporting enzymatic activities of measles and Nipah virus L polymerases. J Virol 90:6642–6656. doi:10.1128/JVI.00602-16 27170753 PMC4944277

[43] Katoh H, Kubota T, Nakatsu Y, Tahara M, Kidokoro M, Takeda M. 2017. Heat shock protein 90 ensures efficient mumps virus replication by assisting with viral polymerase complex formation. J Virol 91:e02220-16. doi:10.1128/JVI.02220-16 28053100 PMC5331814

[44] Connor JH, McKenzie MO, Parks GD, Lyles DS. 2007. Antiviral activity and RNA polymerase degradation following Hsp90 inhibition in a range of negative strand viruses. Virology (Auckl) 362:109–119. doi:10.1016/j.virol.2006.12.026 PMC1995422 17258257

[45] Grard G, Fair JN, Lee D, Slikas E, Steffen I, Muyembe J-J, Sittler T, Veeraraghavan N, Ruby JG, Wang C, Makuwa M, Mulembakani P, Tesh RB, Mazet J, Rimoin AW, Taylor T, Schneider BS, Simmons G, Delwart E, Wolfe ND, Chiu CY, Leroy EM. 2012. A novel rhabdovirus associated with acute hemorrhagic fever in central Africa. PLoS Pathog 8:e1002924. doi:10.1371/journal.ppat.1002924 23028323 PMC3460624

[46] McMorris FA, Ruddle FH. 1974. Expression of neuronal phenotypes in neuroblastoma cell hybrids. Dev Biol 39:226–246. doi:10.1016/0012-1606(74)90237-1 4851982

[47] Ito N, Takayama‐Ito M, Yamada K, Hosokawa J, Sugiyama M, Minamoto N. 2003. Improved recovery of rabies virus from cloned cDNA using a vaccinia virus‐free reverse genetics system. Microbiol Immunol 47:613–617. doi:10.1111/j.1348-0421.2003.tb03424.x 14524622

[48] Kobasa D, Rodgers ME, Wells K, Kawaoka Y. 1997. Neuraminidase hemadsorption activity, conserved in avian influenza A viruses, does not influence viral replication in ducks. J Virol 71:6706–6713. doi:10.1128/JVI.71.9.6706-6713.1997 9261394 PMC191950

[49] Masatani T, Ito N, Shimizu K, Ito Y, Nakagawa K, Sawaki Y, Koyama H, Sugiyama M. 2010. Rabies virus nucleoprotein functions to evade activation of the RIG-I-mediated antiviral response. J Virol 84:4002–4012. doi:10.1128/JVI.02220-09 20130065 PMC2849511

[50] Minamoto N, Tanaka H, Hishida M, Goto H, Ito H, Naruse S, Yamamoto K, Sugiyama M, Kinjo T, Mannen K, Mifune K. 1994. Linear and conformation‐dependent antigenic sites on the nucleoprotein of rabies virus. Microbiol Immunol 38:449–455. doi:10.1111/j.1348-0421.1994.tb01806.x 7526134

[51] Yamaoka S, Ito N, Ohka S, Kaneda S, Nakamura H, Agari T, Masatani T, Nakagawa K, Okada K, Okadera K, Mitake H, Fujii T, Sugiyama M. 2013. Involvement of the rabies virus phosphoprotein gene in neuroinvasiveness. J Virol 87:12327–12338. doi:10.1128/JVI.02132-13 24027304 PMC3807887

[52] Ito N, Okamoto T, Sasaki M, Miyamoto S, Takahashi T, Izumi F, Inukai M, Jarusombuti S, Okada K, Nakagawa K, Fujii Y, Nishiyama S, Masatani T, Sawa H, Sugiyama M. 2021. Safety enhancement of a genetically modified live rabies vaccine strain by introducing an attenuating Leu residue at position 333 in the glycoprotein. Vaccine (Auckl) 39:3777–3784. doi:10.1016/j.vaccine.2021.05.002 34092430

[53] Pettersen EF, Goddard TD, Huang CC, Meng EC, Couch GS, Croll TI, Morris JH, Ferrin TE. 2021. UCSF ChimeraX: structure visualization for researchers, educators, and developers. Protein Sci 30:70–82. doi:10.1002/pro.3943 32881101 PMC7737788

