# Supplementary material for: Functional dissection of the C-terminal domain of rabies virus RNA polymerase L protein
Source: J Virol. 2025 Mar 11;99(4):e02082-24. doi: 10.1128/jvi.02082-24 (PMC11998541; doi:10.1128/jvi.02082-24)
Supplement: Fig. S1 and S2 — Alignments of amino acid sequences of lyssavirus L and P proteins. [file jvi.02082-24-s0001.pdf]

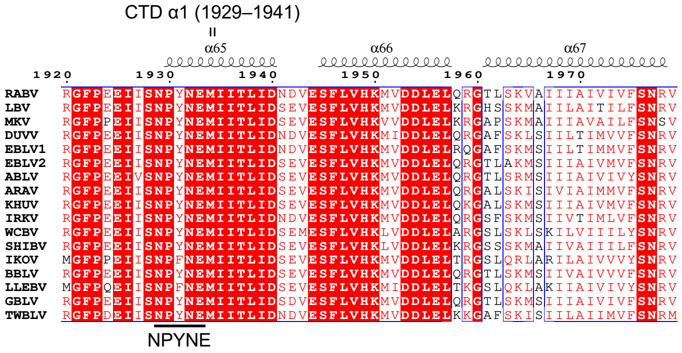

Fig. S1. Comparison of the amino acid sequences of the L proteins of lyssavirus species. Complete amino acid sequences of the L proteins from the 17 lyssavirus species were obtained from the GenBank database. Multiple sequence alignment was performed using MAFFT version 7 (1) and presented using ESPript 3.0 (2). Amino acid residues 1920–1979 of the L protein are shown with the secondary structure based on the structure of the RABV L protein (PDB ID: 6UEB). The 65th α-helix in the L protein (α65) corresponds to the CTD α1 (amino acid residues 1929–1941). Location of the NPYNE sequence at L1929–1933 is indicated below the alignment. Completely conserved residues are indicated in white with a red background. Highly conserved residues (similarity rate of ≥ 80%) and their biochemically similar residues are shown in red within blue frames. RABV, RABV-wt (GenBank accession number: AB044824); LBV, Lagos bat lyssavirus (GenBank accession number: EU293110); MKV, Mokola lyssavirus (GenBank accession number: NC_006429); DUVV, Duvenhage lyssavirus (GenBank accession number: EU293119); EBLV1, European bat lyssavirus 1 (GenBank accession number: NC_009527); EBLV2, European bat lyssavirus 2 (GenBank accession number: NC_009528); ABLV, Australian bat lyssavirus (GenBank accession number: NC_003243); ARAV, Aravan lyssavirus (GenBank accession number: EF614259); KHUV, Khujand lyssavirus (GenBank accession number: EF614261); IRKV, Irkut lyssavirus (GenBank accession number: EF614260); WCBV, West Caucasian bat lyssavirus (GenBank accession number: EF614258); SHIBV, Shimoni bat lyssavirus (GenBank accession number: GU170201); IKOV, Ikoma lyssavirus (GenBank accession number: NC_018629); BBLV, Bokeloh bat lyssavirus (GenBank accession number: NC_025251); LLEBV, Lleida bat lyssavirus (GenBank accession number: NC_031955); GBLV, Gannoruwa bat lyssavirus (GenBank accession number: NC_031988); TWBLV, Tiwan bat lyssavirus (GenBank accession number: NC_055474).

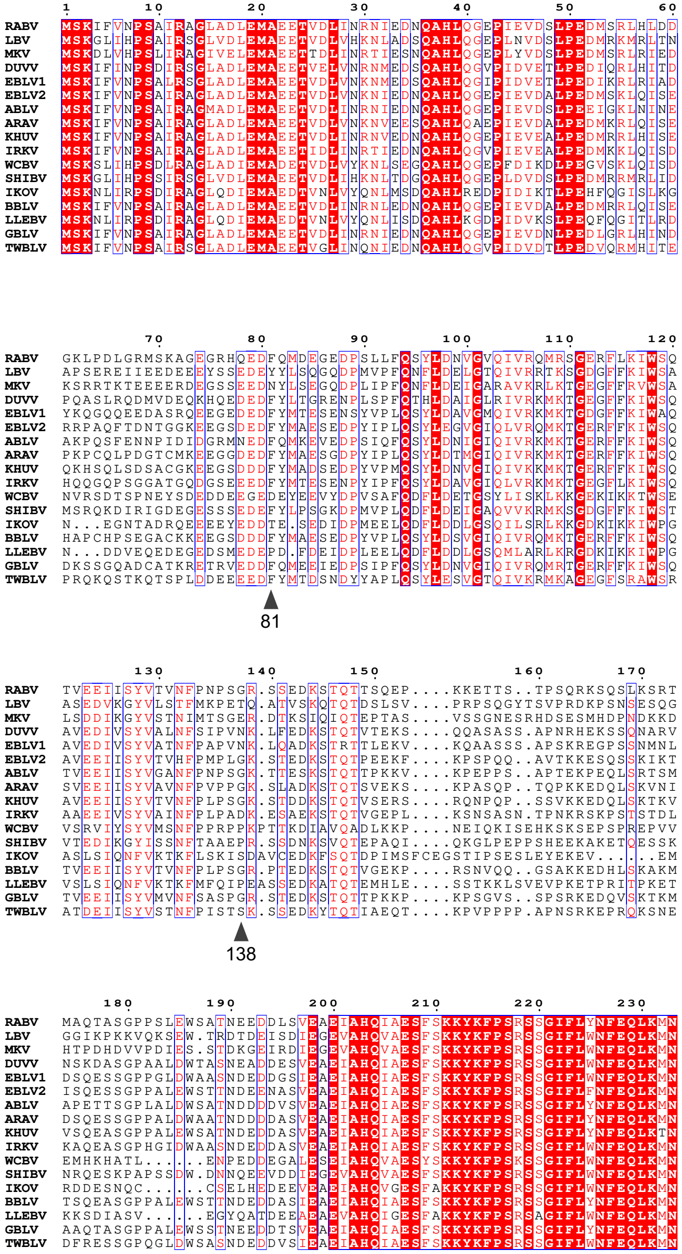

Fig S2
Izumi *et al.*

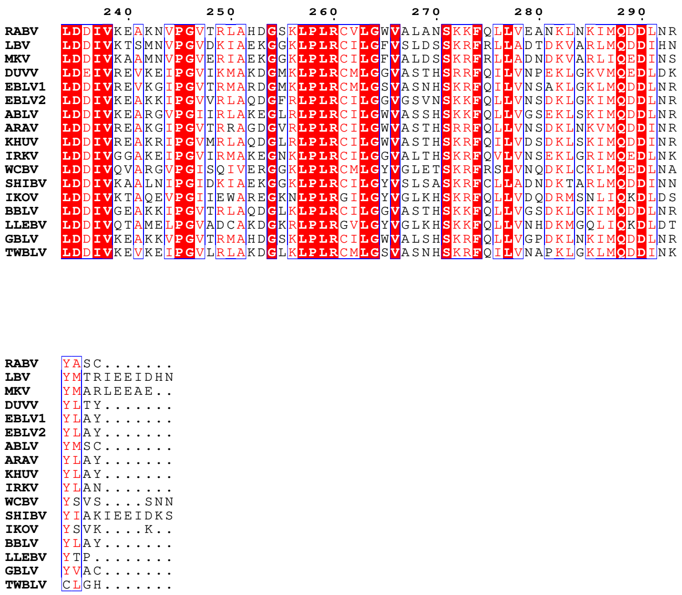

Fig. S2. Comparison of the amino acid sequences of the P proteins of lyssavirus species. Complete amino acid sequences of the P proteins from the 17 lyssavirus species were obtained from the GenBank database. Multiple sequence alignment was performed using MAFFT version 7 (1) and presented using ESPript 3.0 (2). The black triangles indicate the residues at P81/138. Completely conserved residues are indicated in white with a red background. Highly conserved residues (similarity rate of ≥ 80%) and their biochemically similar residues are shown in red within blue frames. RABV, RABV-wt (GenBank accession number: AB044824); LBV, Lagos bat lyssavirus (GenBank accession number: EU293110); MKV, Mokola lyssavirus (GenBank accession number: NC_006429); DUVV, Duvenhage lyssavirus (GenBank accession number: EU293119); EBLV1, European bat lyssavirus 1 (GenBank accession number: NC_009527); EBLV2, European bat lyssavirus 2 (GenBank accession number: NC_009528); ABLV, Australian bat lyssavirus (GenBank accession number: NC_003243); ARAV, Aravan lyssavirus (GenBank accession number: EF614259); KHUV, Khujand lyssavirus (GenBank accession number: EF614261); IRKV, Irkut lyssavirus (GenBank accession number: EF614260); WCBV, West Caucasian bat lyssavirus (GenBank accession number: EF614258); SHIBV, Shimoni bat lyssavirus (GenBank accession number: GU170201); IKOV, Ikoma lyssavirus (GenBank accession number: NC_018629); BBLV, Bokeloh bat lyssavirus (GenBank accession number: NC_025251); LLEBV, Lleida bat lyssavirus (GenBank accession number: NC_031955); GBLV, Gannoruwa bat lyssavirus (GenBank accession number: NC_031988); TWBLV, Tiwan bat lyssavirus (GenBank accession number: NC_055474).

References

1. Katoh K, Rozewicki J, Yamada KD. 2019. MAFFT online service: multiple sequence alignment, interactive sequence choice and visualization. Brief Bioinform 20:1160–1166.

2. Robert X, Gouet P. 2014. Deciphering key features in protein structures with the new ENDscript server. Nucleic Acids Res 42:W320-324.
